# Search for the glycated hemoglobin target minimizing mortality among people with type 2 diabetes with rare incidence of treatment-related severe hypoglycemia – Epidemiologic analysis of J-DOIT3 randomized controlled trial

**DOI:** 10.1371/journal.pone.0360295

**Published:** 2026-10-23

**Authors:** Mitsuhiko Noda, Kohjiro Ueki, Takayoshi Sasako, Naoki Ishizuka, Kana Miyake, Kengo Miyoshi, Tomohisa Aoyama, Yukiko Okazaki, Yasuo Ohashi, Toshimasa Yamauchi, Takashi Kadowaki, The J-DOIT3 Study Group

**Affiliations:** 1 Department of Diabetes, Metabolism and Endocrinology, Ichikawa Medical Center, International University of Health and Welfare, Chiba, Japan; 2 Department of Endocrinology and Diabetes, Saitama Medical University, Saitama, Japan; 3 Diabetes Research Center, Japan Institute for Health Security, Tokyo, Japan; 4 Department of Diabetes and Metabolic Diseases, Graduate School of Medicine, The University of Tokyo, Tokyo, Japan; 5 Okachimachi Ohisama Clinic, Tokyo, Japan; 6 Center for Digital Transformation for Health, Kyoto University School of Medicine, Kyoto, Japan; 7 Department of Metabolism and Endocrinology, Juntendo University Graduate School of Medicine, Tokyo, Japan; 8 Department of Integrated Science and Engineering for Sustainable Society, Chuo University, Tokyo, Japan; 9 Toranomon Hospital, Tokyo, Japan; Japanese Academy of Health and Practice, JAPAN

## Abstract

**Objectives:**

In recent years, with the considerable changes in the therapeutic armamentarium for diabetes, the incidence of hypoglycemia events, especially severe hypoglycemia events, associated with drug treatment in people with diabetes has markedly decreased. We were prompted to evaluate the threshold for each intervention target, especially glycemic control, associated with a minimized risk of the macrovascular complications of diabetes in this setting.

**Methods:**

In the Japan Diabetes Optimal Integrated Treatment study for 3 major risk factors of cardiovascular diseases (J-DOIT3) conducted by us previously, we recruited 2542 participants and assigned them to a conventional therapy group (n = 1271) and an intensive therapy group (n = 1271; two ineligible participants were removed from all analyses afterwards), and the incidence of severe hypoglycemia events was low in both groups (7 in the intensive therapy group and 4 in the conventional therapy group during a median 8.5 years’ follow-up). Towards the objective of this study, we undertook a landmark analysis with a landmark time-point of 1 year after the baseline of the J-DOIT3 study. Then, using the updated mean values of the glycated hemoglobin (HbA1c), systolic blood pressure, and LDL-cholesterol and HDL-cholesterol levels, we determined the optimal levels of each of these variables associated with the lowest hazard ratios for outcomes, including the primary and cardiovascular outcomes.

**Results:**

For glycemic control, our results revealed no clear lower threshold of the glycated hemoglobin associated with the lowest risk of myocardial infarction or all-cause mortality; for other risk factors, it might be an important treatment target to maintain the LDL-cholesterol levels at below 80 mg/dL to minimize the risk of stroke.

**Conclusions:**

The present analysis suggested that the treatment target for HbA1c to minimize the risk of all-cause mortality in participants with diabetes with a low incidence of severe hypoglycemia episodes could be < 6.5% (ClinicalTrials.gov, number NCT00300976).

## Introduction

Glucose-lowering therapy is the mainstay of treatment for diabetes. However, no clear conclusions have been drawn so far about the optimal levels of glycemic control. In the ACCORD trial [1] that started in 2001, the main objective was strict glucose control in the intensive glycemia control group, but this resulted in a significant increase in the mortality rate unexpectedly observed in this group as compared with that in the standard glycemic target group, so that the study was suspended. Although no conclusive reason was identified for the higher mortality in the intensive glycemia control group, it is speculated that severe hypoglycemia (hypoglycemia requiring third party assistance or hospitalization) may have played some role in the increased rate of mortality by potentially increased undetected cardiovascular events [2]. In fact, the results of a meta-analysis [3] conducted with 6 studies including data from the ADVANCE trial [4] and the VADT [5], which were implemented under a similar two-treatment arms (intensive or standard) protocol to that in the ACCORD trial, suggested that severe hypoglycemia may well be associated with a higher risk of cardiovascular disease in people with diabetes. In the Steno-2 study, in which 80 participants were assigned to the intensive treatment arm and received multifactorial interventions, a significant reduction in both macrovascular and microvascular outcomes was observed compared with those 80 participants assigned to the conventional treatment arm [6], and the investigators claimed that lipid-lowering therapy played a major role in the reduced incidence of cardiovascular events [7].

For the Japan Diabetes Optimal Integrated Treatment study for 3 major risk factors of cardiovascular diseases (J-DOIT3) conducted by us previously [8], we recruited 2542 participants and assigned them to a conventional therapy group (n = 1271) and intensive therapy group (n = 1271; two ineligible participants were removed from all analyses afterwards), and the latter group received multifactorial interventions for control of blood glucose, blood pressure, and lipids [8]. The study protocol is described in detail elsewhere [9]. In the intensive therapy group, we observed a non-significant reduction (HR 0.81, 95% CI 0.63–1.04; p = 0.094) in the incidence of the primary outcome events comprised of the occurrence of myocardial infarction, stroke (cerebral infarction, cerebral hemorrhage, or subarachnoid hemorrhage), all-cause mortality, and revascularization as compared with that in the conventional therapy arm [8]; however, the difference became significant (HR 0.76, 0.59–0.99; p = 0.042) after prespecified adjustment for the baseline risk factors. Especially for cerebrovascular events, which were stroke in almost all cases, the difference between the two groups was pronounced (HR 0.42, 95% CI 0.24–0.74; p = 0.002; before adjustment). It is worth noting that severe hypoglycemic events were rare in both groups, even in the intensive therapy group (7 episodes in the intensive therapy group and 4 episodes in the conventional therapy group over the study period, namely, during the median 8.5 years’ follow-up) [8].

Glucose-lowering therapy for diabetes has changed significantly in recent years, *e.g.*, the use of insulin monotherapy and sulfonylureas has greatly decreased [10]. Therefore, the incidence of severe hypoglycemic events has also decreased considerably as compared with that in the era of the ACCORD and ADVANCE trials, and the VADT. Given this infrequent occurrence of severe hypoglycemia, we considered it important to determine a lower glycemia threshold for minimizing the risk of diabetes-related outcomes, including cardiovascular events in such a setting. Therefore, we reviewed and analyzed the data of J-DOIT3, in which, as described above, the incident rate of severe hypoglycemia episodes requiring third-party assistance was extremely low in both the treatment arms; *i.e.*, around 0.03 and 0.02 per 100 person-years for the intensive and conventional therapy groups, respectively. In fact, regarding the ACCORD trial, there were 3.0 and 1.0 serious hypoglycemic events per 100 person-years for the intensive and standard glucose control, respectively [11], and in the ADVANCE trial, the incident rate of severe hypoglycemia was 0.7 per 100 person-years in the group receiving intensive glucose control and 0.4 in the standard group. In the VADT, the incident rate of severe hypoglycemia in the intensive treatment group was 10.3 per 100 person-years compared with 3.7 per 100 person-years in the standard treatment group [12]. These incident rates of the 3 trials were about 20 to more than 100 times higher than experienced in the J-DOIT3.

Furthermore, prescription of newer drugs, *e*.*g*., DPP-4 inhibitors, GLP-1 receptor agonists, and SGLT2inhibitors, which are less likely to cause hypoglycemia, has expanded even further after the J-DOIT3 study [13], which would make our current analysis even more meaningful.

We performed the post-hoc analyses using updated mean values, which were also utilized in the UK Prospective Diabetes Study [14,15], of each variable of the intervention target in the J-DOIT3: namely, glycated hemoglobin (HbA1c), systolic blood pressure (SBP), and LDL-cholesterol. In addition, we also included HDL-cholesterol, which showed a significant difference between the two treatment arms [8], in this analysis as an explanatory variable. In this way, we attempted to precisely determine the dynamic effect range of each parameter for specific outcomes. In other words, we attempted to identify the thresholds of HbA1c and other clinical determinants associated with a minimized risk of macrovascular complications of diabetes, especially isolated outcomes, *e.g.*, stroke, myocardial infarction, and mortality, in the setting of the J-DOIT3 trial.

It should be noted here that detailed results and discussions regarding renal outcomes—which are no less important than cardiovascular outcomes in diabetic vascular complications—have already been reported in our previous publication [16].

## Methods

### Study design

In the multicenter, open-label, randomized, parallel-group J-DOIT3 trial, which was undertaken with the participation of 81 institutions in Japan, we examined the effectiveness of intensive multifactorial therapy in preventing the development and progression of diabetic complications in people with type 2 diabetes (ClinicalTrials.gov identifier: NCT00300976; clinicaltrials.gov/study/NCT00300976; first registered on March 10, 2006). The study rationale and design, as well as the multifactorial interventions employed, have been reported previously (Fig 1) [8,9].

**Fig 1:**
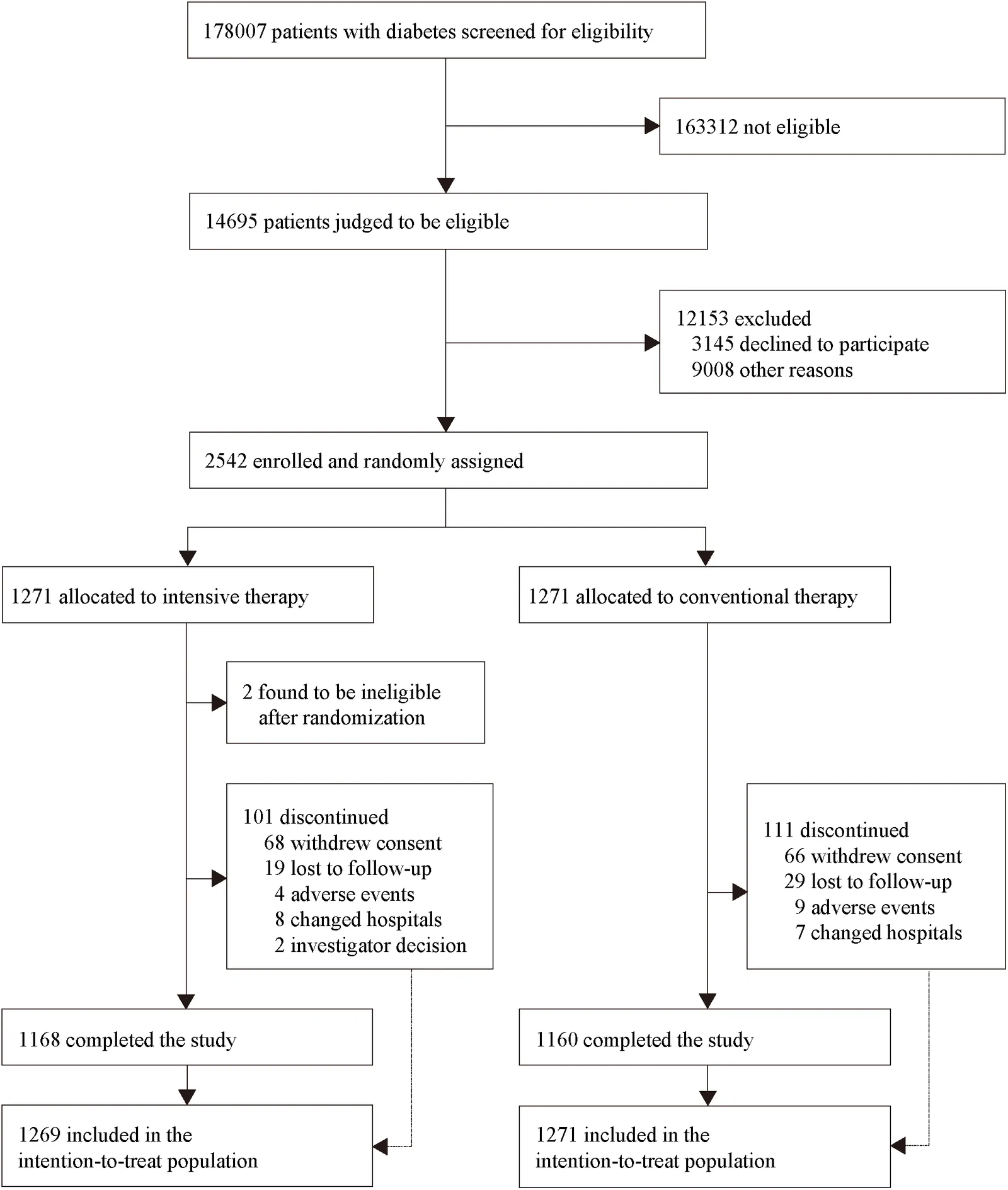
Enrollment, randomization and follow-up of participants. Two patients in the intensive therapy group were turned out to be ineligible because one matched the exclusion criteria in low-density lipoprotein cholesterol level and the other did not match the inclusion criteria in medication at baseline immediately after randomization. Both were excluded from the entire study.

From the participant institutions that had diabetes care clinics with diabetes specialists and educators, we recruited participants with type 2 diabetes aged 45–69 years old with hypertension and/or dyslipidemia and HbA1c values of 6.9% or higher who were receiving treatment with the following: diet and exercise alone; diet and exercise therapy plus 1 oral antidiabetic drug; or diet and exercise therapy plus an α-glucosidase inhibitor and another oral antidiabetic drug, between June 16, 2006, and March 31, 2009. Details of the inclusion and exclusion criteria are described in the Appendix in the online supplemental file. The study protocol was approved by the ethics committee of each of the participating institutions. Written informed consent for participation was obtained from all the participants. The study was carried out in accordance with the principles of the Declaration of Helsinki as revised in 2008.

The participants were randomly assigned at a 1:1 ratio by the dynamic balancing minimization method [17], using sex, age, glycated hemoglobin level, and history of cardiovascular disease as stratification factors to receive conventional therapy for control of HbA1c, BP, and lipids as specified in the Japanese guidelines at that time (HbA1c < 6.9%; BP < 130/80 mm Hg; LDL-C < 120 mg/dl or 100 mg/dl in those with a history of cardiovascular disease; HDL-C ≥ 40 mg/dl; and triglycerides <150 mg/dl) [18], or intensive therapy with stricter targets (HbA1c < 6.2%; BP < 120/75 mm Hg; LDL-C < 80 mg/dl or 70 mg/dl in those with a history of cardiovascular disease; HDL-C ≥ 40 mg/dl; and triglycerides <120 mg/dl).

Assignments were done by the electronic data capturing system with computer-generated random numbers and a computer minimization program. Computer programs were developed for the intended analyses, which followed the prespecified statistical analysis plan, with the investigators blinded to the treatment assignment of the participants.

### Treatment procedure

Participants in the conventional therapy group were treated in accordance with the Japanese guidelines at that time [18], which recommended the same HbA1c, BP, and LDL-C targets adopted in this trial. For glycemic control, there was no algorithm for the start of medication, but it had to be chosen in combination with diet and exercise, taking into account the participant’s pathogenesis, age, social environment, and such. For BP control, treatment was started with an angiotensin II-receptor blocker, angiotensin-converting enzyme inhibitor, or Ca channel blocker as the first-line treatment if the target BP was not achieved with lifestyle modification alone. For LDL-C control, use of a statin was recommended if the target LDL-C level was not achieved with therapeutic diet modification alone.

In the intensive therapy group, the treatment intensification procedures were as follows. As for glycemic control, the preregistration antidiabetic therapy, along with the predetermined diet and exercise therapy, was maintained in all participants. If a glycated hemoglobin level of <6.2% or a > 1.0% reduction of the glycated hemoglobin failed to be achieved within 3 months, treatment was intensified in steps using antidiabetic drugs from 4 classes, as described in a previous report [9], and shown in S1 Table. All participants in the intensive therapy group were also given a blood glucose meter for self-monitoring.

For BP control, the preregistration hypertension treatment, along with the predetermined diet and exercise therapy, was maintained in all the participants. If the BP, as measured at a clinic by the physician in charge or other health care provider (single measurement) after 3 months was > 120/75 mm Hg, additional antihypertensive drug therapy was prescribed in steps (first-line treatment: angiotensin II–receptor blocker or angiotensin-converting–enzyme inhibitor) to ensure that the treatment goal was achieved within 3–6 months, as described previously and shown in S1 Table [9]. All participants in the intensive therapy group received a sphygmomanometer at study entry.

For control of the lipids, the preregistration lipid-lowering treatment, along with diet and exercise therapy, was maintained in all the participants. If the lipid control goal was not achieved within 3 months, further lipid-lowering drugs were prescribed (first-line treatment: high-intensity statin), as described previously and shown in S1 Table [9].

### Outcomes

The incidence rate of severe hypoglycemia was screened from the trial data, and the relationships between trial outcomes were assessed.

The primary outcome was the occurrence of any of a composite of macrovascular events, as mentioned in the following paragraph, and all-cause mortality, as assessed by an Endpoint Assessment Committee that was unaware of group assignment.

The macrovascular events were defined as myocardial infarction, coronary artery bypass surgery, percutaneous transluminal coronary angioplasty, stroke, carotid endarterectomy, percutaneous transluminal cerebral angioplasty, and carotid artery stenting (the information is given in greater detail in the previous report of the trial results) [9].

The main secondary outcome was a composite of myocardial infarction, stroke, and all-cause mortality, which was the original primary outcome and changed to the main secondary outcome when the protocol was modified and approved in 2010 [9].

### Statistical procedure

Time-dependent landmark analyses were undertaken using updated mean values of each of the relevant variables, namely, HbA1c, SBP, and LDL-cholesterol, with a landmark time-point of 1 year after the baseline of the study, a time point at which we assumed the sequelae of the pre-baseline characteristics would have diminished sufficiently, and the effects of the interventions might have reached a steady state.

We also included HDL-cholesterol, which showed a significant difference between the two arms, in this analysis as an explanatory variable [8].

The hazard ratios for potential background risk factors were estimated by Cox regression analysis. Statistical significance was set at a two-sided p-value of <0.05.

### Role of the funding source

The J-DOIT3 trial was investigator-initiated. The funder of the study had no role in study design, data collection, data analysis, data interpretation, or writing of the report. All study data were collected and retained by the investigators and were not made available to the funding sources. The corresponding authors had full access to all the data in the study and had final responsibility for the decision to submit for publication.

## Results

### Participants’ characteristics

Table 1 shows the characteristics of the participants from both the conventional and intensive therapy arms and those of the participants of the study baseline. Although 30 participants from the intensive therapy group (including 23 who withdrew their consent, 2 who were lost to follow-up, 2 who developed adverse events, 1 who changed hospitals, and 2 at the discretion of the investigator) and 24 participants from the conventional therapy group (including 2 who died, 16 who withdrew consent, 2 who were lost to follow-up, 3 who developed adverse events, and 1 who changed hospitals) were omitted from the original cohort [8], the characteristics of the participants analyzed in this time-dependent landmark analysis were basically similar to those of the participants in the original study; The participants’ characteristics were similar between the two study groups, except for the difference in the smoking status, with a higher proportion of current smokers in the intensive therapy group than in the conventional therapy group. One year after the landmark time-point, the mean (standard deviation) values of HbA1c, SBP, and LDL-cholesterol were 7.0% (0.9%), 125.3 (14.3) mmHg, and 97.3 (28.5) mg/dL, respectively; the mean (SD) values of HDL-cholesterol were 56.1 (15.5) mg/dL for men and 62.3 (15.6) mg/dL for women. The time-course of these parameters after 1 year is shown in S2 Table with the landmark time-point data.

**Table 1:** Baseline characteristics of the analyzed participants for the time-dependent landmark analysis with a landmark time-point of 1 year.

	Conventional therapy (n = 1247)	Intensive therapy (n = 1239)	Total (n = 2486)	P value between the two groups
Age (years)	59.1 (6.3)	58.9 (6.4)	59.0 (6.3)	0.527
Sex				0.771
Men	776 (62.2%)	764 (61.7%)	1540 (61.9%)	
Women	471 (37.8%)	475 (38.3%)	946 (38.1%)	
Duration of diabetes (years)	8.52 (7.01)	8.61 (7.00)	8.57 (7.00)	0.732
Smoking status				**0.014**
Never	576 (46.2%)	559 (45.1%)	1135 (45.7%)	
Former	410 (32.9%)	363 (29.3%)	773 (31.1%)	
Current	261 (20.9%)	317 (25.6%)	578 (23.3%)	
History of cardiovascular disease	141 (11.3%)	143 (11.5%)	284 (11.4%)	0.854
Bodyweight (kg)	65.81 (12.01)	65.38 (11.90)	65.59 (11.95)	0.372
BMI (kg/m^2^)	24.9 (3.8)	24.8 (3.5)	24.8 (3.7)	0.458
Fasting plasma glucose (mg/dL)	158.8 (39.3)	159.5 (41.5)	159.1 (40.4)	0.681
HbA1c (%)	8.0 (1.0)	8.0 (1.0)	8.0 (1.0)	0.462
Systolic blood pressure (mm Hg)	134.1 (16.3)	133.7 (16.8)	133.9 (16.6)	0.578
Diastolic blood pressure (mm Hg)	80.0 (11.2)	79.4 (10.8)	79.7 (11.0)	0.178
LDL cholesterol (mg/dL)	125.8 (31.8)	125.3 (30.5)	125.5 (31.2)	0.687
HDL cholesterol (mg/dL)	54.7 (14.0)	54.3 (15.0)	54.5 (14.5)	0.587
Triglycerides (mg/dL)	147.8 (98.7)	148.4 (104.6)	148.1 (101.7)	0.881

Mean (SD) or number (%).

This table presents baseline data—the data collected at enrollment—for participants in the J-DOIT3 study included in this time-dependent landmark analysis. The characteristics were similar between the two groups except for smoking status. The t-test and the χ2-test were used for continuous and categorical variables, respectively.

Bold indicates significant p-values.

### Number of outcomes after the landmark time-point

In total, 223 primary outcome events (103 in the intensive therapy and 120 in the conventional therapy group) occurred during the term of the landmark analysis. Among these 150 (67 and 83 in the intensive and conventional therapy groups, respectively) were the main secondary outcomes, including myocardial infarction of 14 events (4 and 10 events in the intensive and conventional therapy groups, respectively), stroke of 41 events (12 and 29 in the intensive and conventional therapy groups, respectively), and death in 86 cases (46 and 40 in the intensive and conventional therapy groups, respectively); total 9 outcome events occurred after revascularization, including myocardial infarction of 2 events, stroke of 3 events, and death in 4 cases. A detailed list of the outcomes is provided in Table 2.

**Table 2:** Number of outcome events in the intensive and conventional therapy groups after the landmark time-point for time-dependent analyses.

	Conventional therapy (n = 1247)	Intensive therapy (n = 1239)	Total (n = 2486)
Primary	120 (9.6%)	103 (8.3%)	223 (9.0%)
Main secondary	83 (6.7%)	67 (5.4%)	150 (6.0%)
**Myocardial infarction**	10 (0.8%)	4 (0.3%)	14 (0.6%)
Coronary artery bypass surgery	5 (0.4%)	5 (0.4%)	10 (0.4%)
Percutaneous transluminal coronary angioplasty	32 (2.6%)	35 (2.8%)	67 (2.7%)
**Cerebral infarction**	26 (2.1%)	10 (0.8%)	36 (1.4%)
**Cerebral hemorrhage**	2 (0.2%)	2 (0.2%)	4 (0.2%)
**Subarachnoid hemorrhage**	1 (0.1%)	0 (0.0%)	1 (0.0%)
Cerebral revascularization	4 (0.3%)	1 (0.1%)	5 (0.2%)
**All-cause mortality**	40 (3.2%)	46 (3.7%)	86 (3.5%)

Data are numbers (percentages).

The primary outcome was a composite of coronary events, cerebrovascular events, and all-cause mortality. The main secondary outcome was a composite of myocardial infarction, stroke, and all-cause mortality.

The bolded item indicates each outcome other than revascularization.

A total 9 main secondary outcome events occurred after revascularization, including myocardial infarction in 2 events in the intensive therapy group, stroke in 3 events (1 and 2 events in the intensive and conventional therapy groups, respectively), and death in 4 cases (2 and 2 in the intensive and conventional therapy groups, respectively).

### Time-dependent landmark analysis

We performed epidemiological analysis for each unique isolated outcome (myocardial infarction, stroke, and all-cause mortality) by time-dependent Cox regression analyses in the two intervention categories using updated mean values of each variable of interest. Due to missing data for variables, 75 participants were excluded from the analyses. The specific breakdown is as follows: 62 participants had missing data on duration of diabetes, and 14 had missing data on the urine albumin-to-creatinine ratio. One of these participants had missing data for both measures. As shown in Table 3, for myocardial infarction, a non-significantly smaller hazard ratio of intensive as compared with conventional therapy group (hazard ratio [HR]: 0.628; p = 0.368) was observed in the analysis with prespecified adjustment for the baseline risk factors, which almost disappeared after further adjustment for the updated mean values of HbA1c, SBP, LDL-cholesterol, and HDL-cholesterol (HR: 0.810; p = 0.715); HbA1c (HR: 1.944 for 1%; p = 0.005) and HDL-cholesterol (HR for 10 mg/dL: 0.477; p = 0.004) emerged to be significant. By contrast, for stroke, the significance of the hazard ratio of intensive therapy (HR: 0.429; p = 0.011) was rendered non-significant after the same adjustment; and LDL-cholesterol (HR for 10 mg/dL: 1.198; p = 0.004) was identified as a significant risk factor. For all-cause mortality, the same analysis identified remarkable significance of the glycated hemoglobin (HR for 1%: 1.549; p < 0.001).

**Table 3:** Time-dependent Cox regression analyses for each isolated outcome (myocardial infarction, stroke, and all-cause mortality) regarding intervention categories and the updated mean value of each variable of interest.

	Myocardial infarction (n = 16)		Stroke (n = 43)		All-cause mortality (n = 92)	
	Hazard ratio	P value	Hazard ratio	P value	Hazard ratio	P value
Conv./ Int. group^*1^	0.628	0.368	0.429	**0.011**	1.008	0.970
Conv./ Int. group^*2^	0.810	0.715	0.650	0.280	0.961	0.877
HbA1c (1%)	1.944	**0.005**	1.334	0.129	1.549	**<0.001**
SBP (10 mmHg)	1.000	0.999	1.118	0.379	0.994	0.957
LDL-C (10 mg/dL)	0.942	0.596	1.198	**0.004**	0.907	0.069
HDL-C (10 mg/dL)	0.477	**0.004**	0.781	0.071	0.937	0.406

Hazard ratios for categorical variables are shown with the category to the left of the slash as the referent.

We excluded 75 participants with missing data for variables from a total of 2,411 participants.

Figures in parentheses of the first row indicate the number of outcomes.

Bold indicates significant p-values.

*1: adjusted for baseline risk factors (*i.e.*, stratification variables for allocation factors).

*2: further adjusted for updated mean values of each variable.

In addition, we conducted similar analyses for the composite outcomes, including the primary and main secondary outcomes. For the primary outcome (a composite of coronary events, cerebrovascular events, and all-cause mortality), the non-significant hazard ratio of the intensive therapy group as compared with the conventional therapy (HR: 0.827; p = 0.162) observed after adjustment for prespecified baseline risk factors for the J-DOIT3, decreased after further adjustments for HbA1c, SBP, LDL-cholesterol, and HDL-cholesterol; where HbA1c (HR for 1%: 1.347; p < 0.001) and HDL-cholesterol (0.813 for 10 mg/dL; p < 0.001) emerged to be significant (S3 Table). For the main secondary outcome (a composite of myocardial infarction, stroke, and all-cause mortality), the hazard ratio was almost negated by further adjustment, and HbA1c (HR for 1%: 1.517; p < 0.001) and HDL-cholesterol (HR for 10 mg/dL: 0.821; p = 0.004) were identified as being significant (S3 Table).

### Feasibility of epidemiological procedures and thresholds

As described above, as some variables showed significant associations with the outcomes, we decided to check the threshold effects of these variables by introducing categories for each variable, on the risk-suppressive properties for the outcomes by stratified analysis. The purpose of the current analysis was to identify factors mediating the effects of intervention of the J-DOIT3; because HDL cholesterol levels showed a significant difference between the intensive and conventional therapy groups [8], this variable was included in the analysis. Since there was only a small difference in BMI trends between the two groups, as shown in the same figure, it was not included as an analytical variable. Regarding triglycerides, since we included HDL-cholesterol as described above, triglycerides were omitted, considering the correlation between the two. This was also the case with the procedure that only systolic blood pressure was employed, rather than both systolic and diastolic blood pressure.

First, we calculated the interaction term among the variables (HbA1c, SBP, LDL-cholesterol, and HDL-cholesterol) and intervention arms for the total of five outcomes (myocardial infarction, stroke, and all-cause mortality; primary and main secondary outcome). Among these interaction terms, only the p value between the intervention groups and SBP for the main secondary outcome was slightly smaller than 0.1 (p = 0.097), with all other interaction terms being more than 0.1. Therefore, we conducted a combined analysis for the two intervention groups thereafter.

Table 4 shows the hazard ratios for each stratified variable with the category of the lowest event rate as the referent. For myocardial infarction, lower HbA1c and, in men, higher HDL-cholesterol were significantly associated with reduced hazard ratios; there seemed to be no clear lower threshold for HbA1c. For stroke, lower SBP and lower LDL-cholesterol and, in men, higher HDL-cholesterol were significantly associated with reduced hazard ratios; for LDL-cholesterol, there seemed to be no clear lower threshold. For all-cause mortality, lower HbA1c and, in women, higher HDL-cholesterol were significantly associated with reduced hazard ratios; there seemed to be no clear lower threshold for HbA1c. We performed a sensitivity analysis using a 6-month (180-day) landmark time-point (with 2507 participants) instead of 1 year. However, although there was one death in the latter half of the first year, the data for the variables of this participant were missing, and it was not included in the analysis; there were no cases of myocardial infarction during this period. Therefore, the results remained virtually unchanged (the changes in those values were by far smaller than the smallest digit in the table) for both of these two outcomes. With regard to stroke, seven cases with no missing data were identified during this period; the results are presented in S4 Table. Alteration of the landmark time-point did not show a substantial difference in the stroke, either.

**Table 4:** Hazard ratios with stratified analyses by introducing categories to each variable.

(a) Myocardial infarction							
				Men			
HbA1c (%)	Point estimate	95%CI	P value	HDLC (mg/dL)	Point estimate	95%CI	P value
8.5 ≤	1			<40	1		
7.5 – < 8.5	0.491	0.092–2.626	0.406	40– < 50	0.296	0.079–1.114	0.072
6.5 – < 7.5	0.225	0.045–1.128	0.070	50– < 60	0.223	0.052–0.953	**0.043**
<6.5	0.055	0.004–0.672	**0.023**	60≤	0.127	0.024–0.674	**0.015**
				**Women**			
**SBP (mmHg)**	**Point estimate**	**95%CI**	**P value**	**HDLC (mg/dL)**	**Point estimate**	**95%CI**	**P value**
140≤	1			<40	1		
130 – < 140	1.723	0.325–9.132	0.523	40– < 50	NA	NA	NA
120 – < 130	1.082	0.199–5.886	0.927	50– < 60	NA	NA	NA
<120	0.994	0.170–5.807	0.995	60≤	NA	NA	NA
**LDLC (mg/dL)**	**Point estimate**	**95%CI**	**P value**				
120≤	1						
100 – < 120	1.518	0.371–6.205	0.561				
80 – < 100	0.802	0.154–4.167	0.793				
<80	1.168	0.223–6.122	0.854				
**(b) Stroke**							
				**Men**			
**HbA1c (%)**	**Point estimate**	**95%CI**	**p value**	**HDLC (mg/dL)**	**Point estimate**	**95%CI**	**p value**
8.5 ≤	1			<40	1		
7.5 – < 8.5	0.692	0.214–2.234	0.538	40– < 50	0.615	0.225–1.682	0.344
6.5 – < 7.5	0.464	0.153–1.408	0.175	50– < 60	0.159	0.039–0.641	**0.010**
<6.5	0.557	0.145–2.145	0.395	60≤	0.351	0.111–1.110	0.075
				**Women**			
**SBP (mmHg)**	**Point estimate**	**95%CI**	**p value**	**HDLC (mg/dL)**	**Point estimate**	**95%CI**	**p value**
140≤	1			<40	NA	NA	NA
130 – < 140	0.322	0.117–0.887	**0.028**	40– < 50	1		
120 – < 130	0.491	0.209–1.153	0.103	50– < 60	0.528	0.140–1.991	0.346
<120	0.455	0.181–1.143	0.094	60≤	0.252	0.059–1.073	0.062
**LDLC (mg/dL)**	**Point estimate**	**95%CI**	**p value**				
120≤	1						
100 – < 120	0.529	0.237–1.180	0.120				
80 – < 100	0.432	0.182–1.024	0.057				
<80	0.178	0.047–0.669	**0.011**				
**(c) All-cause mortality**							
				**Men**			
**HbA1c (%)**	**Point estimate**	**95%CI**	**P value**	**HDLC (mg/dL)**	**Point estimate**	**95%CI**	**P value**
8.5 ≤	1			<40	1		
7.5 – < 8.5	0.715	0.298–1.713	0.451	40– < 50	0.852	0.381–1.905	0.696
6.5 – < 7.5	0.395	0.174–0.898	**0.027**	50– < 60	0.538	0.224–1.296	0.167
<6.5	0.282	0.108–0.738	**0.010**	60≤	0.798	0.360–1.771	0.579
				**Women**			
**SBP (mmHg)**	**Point estimate**	**95%CI**	**P value**	**HDLC (mg/dL)**	**Point estimate**	**95%CI**	**P value**
140≤	1			<40	1		
130 – < 140	0.967	0.430–2.172	0.935	40– < 50	0.356	0.077–1.637	0.185
120 – < 130	0.807	0.369–1.764	0.591	50– < 60	0.151	0.032–0.719	**0.018**
<120	1.062	0.486–2.324	0.880	60≤	0.089	0.019–0.421	**0.002**
LDLC (mg/dL)	Point estimate	95%CI	P value				
120≤	1						
100 – < 120	1.018	0.480–2.161	0.962				
80 – < 100	1.430	0.703–2.908	0.323				
<80	1.652	0.776–3.515	0.193				

Hazard ratios for myocardial infarction (a), stroke (b), and all- cause mortality (c) were determined for each stratified variable by time-dependent Cox regression analysis, having the category of the lowest event rate as referent; for HDL-cholesterol, analysis was performed separately for men and women.

Bold indicates significant p-values.

NA: not available because of a small number of events. (a) Because there were only two cases of myocardial infarction among women, the Cox regression failed to converge, and we were unable to estimate the hazard ratio. (b) Since no cases of stroke were observed in women with HDL-C levels <40 mg/dL, this category is designated as NA.

For the primary and main secondary outcomes (S5 Table), the hazard ratios for HbA1c increased from the lowest category (HbA1c < 6.5%); in addition, HDL-cholesterol ≥50 mg/dL (for women) and ≥40 mg/dL (for men) might also be significant determinants of the risk of occurrence of cardiovascular events and death.

In addition, because the HbA1c and HDL-cholesterol showed significant interaction (p = 0.010; chi-square test), we categorized the HDL-cholesterol value into quartiles according to the mean HDL-cholesterol values in the participants up to the third year of follow-up. This sub-analysis revealed that the association between the glycated hemoglobin and all-cause mortality was especially significant in participants with values of HDL-cholesterol in the upper half categories (S6a Table); the same was also the case with cancer mortality (S6b Table).

In addition, as a sensitivity analysis, we extracted and analyzed only participants with no history of cardiovascular events at baseline of the J-DOIT3—that is, the primary prevention cohort (S7 Table). Although the statistical significance of HbA1c to all-cause mortality became non-significant, the overall trend remained unchanged, and an even stronger association was observed between HbA1c and myocardial infarction.

## Discussion

In the present analyses, we estimated the hazard ratios for each treatment target, especially HbA1c, to detect the role of each variable as a clinical determinant of the cardiovascular outcomes and mortality using data from the J-DOIT3 trial in which incidence rate of severe hypoglycemia episodes was very infrequent (6 episodes in the intensive intervention group and 4 episodes in the control group during the landmark analysis term during the median 7.5 years’ follow-up after the landmark time-point). We calculated the interaction term among the variables and intervention arms for the total of five outcomes. Among these interaction terms, only the p value between the intervention groups and SBP for the main secondary outcome was slightly smaller than 0.1 (p = 0.097), with all other interaction terms being more than 0.1. Therefore, we conducted a combined analysis for the two intervention groups thereafter. As shown in Table 4, for myocardial infarction and all-cause mortality, there seemed to be no clear lower threshold for HbA1c; in other words, we did not observe increase in the event rate in the lowest HbA1c category in this relatively old Japanese population (60 years old at the start of the time-dependent analysis). However, we should be very cautious, especially regarding myocardial infarction, because statistically significant differences in the second and third categories might have been overlooked due to the small number of events.

In fact, in the general population, it is well established that the HbA1c levels do not show a J-shaped association with all-cause mortality [19]; by contrast, however, among people with diabetes, most studies have reported J-shaped associations [20–22]. Moreover, among the large-scale randomized controlled trials mentioned in the Introduction section, the ACCORD [23] and ADVANCE [22] trials, but not the UKPDS [14] and VADT [24], revealed a J-shaped relationship with all-cause mortality; the absence of such a relationship in the latter trials could be because of the relatively less strict treatment goals and the smaller number of study participants, respectively.

In these aforementioned trials, around 1%−5% of the participants experienced severe hypoglycemia events [11]. The severe hypoglycemia events could have hampered the analyses and led to difficulty in evaluating the true association between the glycemia level itself and the outcomes independent of severe hypoglycemia.

Although abundant data have been reported on the microvascular complications of diabetes, [1,4–6,14,25,26] few data have been reported on macrovascular complications, especially in relation to the glycemia thresholds. The current epidemiological analyses indicate how treatment goals should be set for HbA1c, blood pressure, LDL-cholesterol, and HDL-cholesterol to minimize the risk of macrovascular complications. In particular, the results of the present analysis might suggest that the possible target HbA1c level to minimize the risk of myocardial infarction (Table 4-a) and all-cause mortality (Table 4-c) could be < 6.5% in a participant population that only rarely experiences severe hypoglycemia episodes. Furthermore, our data suggest that LDL-cholesterol could be a major modifiable factor for preventing stroke, and it might be an important treatment target to maintain the LDL-cholesterol levels at <80 mg/dL.

Increased mortality rates associated with low LDL-cholesterol levels might be like those of the population data [27], in which people with low LDL-cholesterol levels had higher all-cause mortality that was mainly accounted for by excess mortality from cancer, possibly representing higher pre-existing cancer comorbidities of the population who consequently harbored lower LDL-cholesterol levels. Namely, the current association between lower levels of LDL-cholesterol and increased all-cause mortality could be explained by reverse causation, which may be inferred from the number of deaths caused by malignant diseases (S8 Table) that are often associated with decreased cholesterol levels. Conversely, this indicates that with more or less a relatively smaller cardiovascular mortality rate, as compared with some previous studies [22,23], glycemia levels may also have a significant role in cancer survival [28]. In addition, a pronounced association of HbA1c to mortality in the upper half of HDL-cholesterol levels might indicate a relatively high impact of HDL-cholesterol on mortality in its lower range.

Although it is difficult to draw a precise picture regarding gender differences in the correlation between stroke and HDL cholesterol, and between all-cause mortality and HDL cholesterol, our analysis may be in line with the findings that, in the general Japanese population, the correlation with HDL cholesterol tends to be stronger for all-cause mortality among women [29], and for stroke among men [30].

The current analyses had a few strengths. First, in our multifactorial intervention, glycemic control yielded statistically significant results, underscoring the importance of glycemic control for treatment. This appears to be different from the Steno- 2 study in which lipid-lowering therapy rather than glucose-lowering therapy played a major role [7]. Second, because they were based on a vigorously conducted clinical trial, it can be assumed that the retrieved results are based on highly precise data and event rates, which are more robust than those based on real-world data. Third, since this is an analysis of participants with a very low incident rate of severe hypoglycemia events, the conclusion might provide a generalized concept on the relationship between glycemic control and cardiovascular events and death, although given that the category of <6.5% has not been subdivided and the confidence interval is wide, our analysis does not identify the absolute lower limit for safe blood glucose control.

Our results also have some limitations. First, although we enrolled more than 2000 participants, the incidence of outcomes, particularly myocardial infarction, was relatively low. This was mainly due to the high proportion of participants for primary prevention (11.4% as shown in Table 1). This could also explain the absence in the current analyses of the recently proposed U-shaped association of low/high HDL-cholesterol with cardiovascular risk [31,32]. Second, among women, we were unable to fully perform an analysis of the association between HDL-cholesterol and myocardial infarction, as well as a part of stroke, due to the small number of events. Third, the present analysis was conducted on a relatively non-obese Japanese population, and the results may not apply to predominantly obese ethnic groups, but could apply to other Asian populations. Fourth, we excluded 75 participants due to insufficient data for adjustment. Therefore, we are not able to rule out the possibility that this might have introduced some bias. Fifth, in the J-DOIT3 study, only a part of participants in both groups achieved the pre-specified HbA1c treatment targets; in particular, in the intensive therapy group, the mean HbA1c during the intervention period was 6.8%, and only 18% achieved an HbA1c level below the target of 6.2% [8]. This may have masked the occurrence of events at further lower HbA1c levels, potentially resulting in insufficient evaluation of HbA1c at those levels. It is crucial to note that this analysis does not guarantee safety at very low HbA1c levels.

In conclusion, we evaluated the associations of the updated mean values of the modifiable parameters, and also of the partially modifiable HDL-cholesterol, which is in large part genetically determined, with the risk of the macrovascular complications of diabetes and all-cause mortality among participants of the J-DOIT3 trial who had a low incident rate of severe hypoglycemia events. Although these analyses are based on research data completed 10 years ago, we believe they reveal the true and universal impact of a cluster of factors, including glycemic control, on mortality and vascular complications. In fact, this study was completed in March 2016. At that time-point, below 5% of patients had been prescribed GLP-1 receptor agonists at the final visit in both the intensive and conventional therapy groups [8], which was also the case with SGLT2 inhibitors [8]; therefore, it is unlikely that this had a significant impact on the results. Rather, the authors believe that these classes of hypoglycemic agents make it more feasible to achieve glycemic control with fewer episodes of severe hypoglycemia. Our current results encourage people with type 2 diabetes, in that, in the absence of a fear of severe hypoglycemia, they might not need be concerned about lowering their target HbA1c levels to minimize the risk of complications.

## Supporting information

S1 Table Multifactorial stepwise treatment in the intensive therapy group. (DOCX)

S2 Table Time course of each variable of the intervention target and HDL-cholesterol for participants (conventional therapy and intensive therapy groups combined) in the landmark analysis. (DOCX)

S3 Table Time-dependent Cox regression analyses for primary and main secondary outcomes regarding intervention categories and the updated mean value of each variable of interest. (DOCX)

S4 Table Hazard ratios with stratified analyses by introducing categories to each variable with a 6-month (180 days) landmark time-point for stroke; data from 2,507 participants were analyzed. (DOCX)

S5 Table Hazard ratios with stratified analyses by introducing categories to each variable. (DOCX)

S6 Table Stratified analysis of the hazard ratios of HbA1c for all-cause and cancer mortality. (DOCX)

S7 Table Hazard ratios with stratified analyses by introducing categories to each variable among participants with no history of cardiovascular events—that is, the primary prevention cohort. (DOCX)

S8 Table Cause of death. (DOCX)

S1 File Appendix. (DOCX)

S2 File Trial protocol. (DOC)

S3 File CONSORT checklist. (DOCX)
