# Supplementary material for: Search for the glycated hemoglobin target minimizing mortality among people with type 2 diabetes with rare incidence of treatment-related severe hypoglycemia – Epidemiologic analysis of J-DOIT3 randomized controlled trial
Source: PLoS One. 2026 Oct 23;21(10):e0360295. doi: 10.1371/journal.pone.0360295 (PMC13693898; doi:10.1371/journal.pone.0360295)
Supplement: Supplementary file 1 [file pone.0360295.s001.docx]

**S1 Table.** Multifactorial stepwise treatment in the intensive therapy group

|  | Step | Medications ^a^ |
| --- | --- | --- |
| Blood glucose ^b^ | 0 | Diet or exercise (or both) therapy (+ Category D) |
|  | 1 | Category A, if BMI ≥ 25 (+ Category D) |
|  |  | Category A or B, if BMI 22 to < 25 (+ Category D) |
|  |  | Category B or A, if BMI 22 (+ Category D) |
|  | 2 | Category A + B (+ Category D) |
|  | 3 | Category C (+ Category A, B, and D) |
| Blood pressure | 1 | ARB or ACEI, escalated to its maximal dose |
|  | 2 | Maximal dose of ARB or ACEI + a long-acting CCB |
|  | 3 | Maximal dose of ARB or ACEI + a long-acting CCB |
|  |  | + others (a diuretic, β-blockers, and/or α-blocker) |
| Lipid ^c^ | 1 | A standard dose of atorvastatin, pitavastatin, or rosuvastatin |
|  | 2 | Maximal dose of atorvastatin, pitavastatin, or rosuvastatin |
|  | 3 | Maximal dose of atorvastatin, pitavastatin, or rosuvastatin |
|  |  | + others (an anion-exchange resin and/or ezetimibe) |

ACEI, angiotensin-converting inhibitor; ARB, angiotensin-II-receptor blocker; BMI, body mass index; CCB, calcium channel blocker.

^a^ It is to be left up to the physician in charge how to optimize the medications by taking adverse events (including hypoglycemia and fluid retention) and results of self-monitoring into account.

^b^ Category A: pioglitazone, biguanides, glucagon-like peptide-1 receptor agonists; Category B: sulfonylureas and glinides; Category C: insulin; Category D: α-glucosidase inhibitors, dipeptidyl peptidase-4 inhibitors, sodium-glucose cotransporter-2 inhibitors.

^c^ The omega-3 fatty acids are to be given if the goal for triglycerides has not been achieved. Standard doses of atorvastatin, pitavastatin, and rosuvastatin are 10, 2, and 5 mg, respectively, whereas maximum doses are 20, 4, and 10 mg.
