# Supplementary material for: Search for the glycated hemoglobin target minimizing mortality among people with type 2 diabetes with rare incidence of treatment-related severe hypoglycemia – Epidemiologic analysis of J-DOIT3 randomized controlled trial
Source: PLoS One. 2026 Oct 23;21(10):e0360295. doi: 10.1371/journal.pone.0360295 (PMC13693898; doi:10.1371/journal.pone.0360295)
Supplement: Supplementary file 2 [file pone.0360295.s002.docx]

**S2 Table.** Time course of each variable of the intervention target and HDL-cholesterol for participants (conventional therapy and intensive therapy groups combined) in the landmark analysis

| Years after the landmark time-point | HbA1c (%) | SBP (mmHg) | LDL-cholesterol (mg/dL) | HDL-cholesterol in men (mg/dL) | HDL-cholesterol in women (mg/dL) |
| --- | --- | --- | --- | --- | --- |
| 0 (1) | 7.1±0.9 | 125.2±14.8 | 101.1±30.8 | 56.1±15.3 | 62.8±16.5 |
| 1 (2) | 7.0±0.9 | 125.3±14.3 | 97.3±28.5 | 56.1±15.5 | 62.3±15.6 |
| 2 (3) | 7.0±0.9 | 125.6±13.9 | 96.2±28.5 | 56.6±15.8 | 62.7±15.6 |
| 3 (4) | 7.0±0.8 | 125.4±13.6 | 94.2±27.5 | 56.3±15.4 | 61.7±15.3 |
| 4 (5) | 6.9±0.9 | 125.2±13.7 | 93.1±27.7 | 55.0±15.2 | 61.4±15.4 |
| 5 (6) | 6.9±0.9 | 126.3±14.4 | 91.7±26.8 | 55.3±15.5 | 61.5±16.2 |
| 6 (7) | 7.0±0.9 | 126.3±14.3 | 91.2±27.4 | 55.0±14.9 | 61.0±15.8 |
| 7 (8) | 6.9±0.9 | 126.7±14.1 | 89.8±26.3 | 54.3±15.1 | 61.6±15.4 |
| 8 (9) | 6.9±0.9 | 125.5±14.0 | 91.2±27.3 | 56.1±15.0 | 61.5±14.6 |

The numbers in the parentheses indicate years after baseline.
