# Supplementary material for: Search for the glycated hemoglobin target minimizing mortality among people with type 2 diabetes with rare incidence of treatment-related severe hypoglycemia – Epidemiologic analysis of J-DOIT3 randomized controlled trial
Source: PLoS One. 2026 Oct 23;21(10):e0360295. doi: 10.1371/journal.pone.0360295 (PMC13693898; doi:10.1371/journal.pone.0360295)
Supplement: Supplementary file 3 [file pone.0360295.s003.docx]

**S3 Table.** Time-dependent Cox regression analyses for primary and main secondary outcomes regarding intervention categories and the updated mean value of each variable of interest

|  | Primary (n = 223) | | Main secondary (n = 150) | |
| --- | --- | --- | --- | --- |
|  | Hazard ratio | P value | Hazard ratio | P value |
| Conv. / Int. group^*1^ | 0.827 | 0.162 | 0.776 | 0.128 |
|  |  |  |  |  |
| Conv. / Int. group^*2^ | 1.064 | 0.704 | 0.949 | 0.794 |
| HbA1c (1%) | 1.347 | **<0.001** | 1.517 | **<0.001** |
| SBP (10 mmHg) | 1.062 | 0.319 | 1.043 | 0.578 |
| LDL-C (10 mg/dL) | 1.044 | 0.155 | 1.011 | 0.776 |
| HDL-C (10 mg/dL) | 0.813 | **<0.001** | 0.821 | **0.004** |

Hazard ratios for categorical variables are shown with the category to the left of the slash as the referent.

We excluded 75 patients with missing data for variables from a total of 2,411 subjects.

Figures in parentheses of the first row indicate the number of outcomes.

Bold indicates significant p-values.

*1: adjusted for baseline risk factors (*i.e.*, stratification variables for allocation factors).

*2: further adjusted for updated mean values of each variable.
