# Supplementary material for: Search for the glycated hemoglobin target minimizing mortality among people with type 2 diabetes with rare incidence of treatment-related severe hypoglycemia – Epidemiologic analysis of J-DOIT3 randomized controlled trial
Source: PLoS One. 2026 Oct 23;21(10):e0360295. doi: 10.1371/journal.pone.0360295 (PMC13693898; doi:10.1371/journal.pone.0360295)
Supplement: Supplementary file 4 [file pone.0360295.s004.docx]

**S4 Table.** Hazard ratios with stratified analyses by introducing categories to each variable with a 6-month (180 days) landmark time-point for stroke; data from 2,507 participants were analyzed.

|  |  |  |  |  | Men |  |  |  |
| --- | --- | --- | --- | --- | --- | --- | --- | --- |
| HbA1c (%) | Point estimate | 95%CI | P value |  | HDLC (mg/dL) | Point estimate | 95%CI | P value |
| 8.5 ≤ | 1 |  |  |  | <40 | 1 |  |  |
| 7.5 - <8.5 | 0.819 | 0.288 - 2.328 | 0.708 |  | 40 - <50 | 0.441 | 0.176 - 1.106 | 0.081 |
| 6.5 - <7.5 | 0.488 | 0.177 - 1.343 | 0.165 |  | 50 - <60 | 0.206 | 0.067 - 0.635 | **0.006** |
| <6.5 | 0.518 | 0.146 - 1.844 | 0.310 |  | 60≤ | 0.258 | 0.088 - 0.756 | **0.014** |
|  |  |  |  |  | Women |  |  |  |
| SBP (mmHg) | Point estimate | 95%CI | P value |  | HDLC (mg/dL) | Point estimate | 95%CI | P value |
| 140≤ | 1 |  |  |  | <40 | NA | NA | NA |
| 130 - <140 | 0.266 | 0.101 - 0.702 | **0.007** |  | 40 - <50 | 1 |  |  |
| 120 - <130 | 0.439 | 0.201 - 0.960 | **0.039** |  | 50 - <60 | 0.438 | 0.122 - 1.565 | 0.204 |
| <120 | 0.453 | 0.198 - 1.035 | 0.060 |  | 60≤ | 0.277 | 0.077 - 0.995 | **0.049** |
|  |  |  |  |  |  |  |  |  |
| LDLC (mg/dL) | Point estimate | 95%CI | P value |  |  |  |  |  |
| 120≤ | 1 |  |  |  |  |  |  |  |
| 100 - <120 | 0.546 | 0.256 - 1.163 | 0.117 |  |  |  |  |  |
| 80 - <100 | 0.451 | 0.201 - 1.011 | 0.053 |  |  |  |  |  |
| <80 | 0.224 | 0.070 - 0.714 | **0.011** |  |  |  |  |  |
