# Supplementary material for: Search for the glycated hemoglobin target minimizing mortality among people with type 2 diabetes with rare incidence of treatment-related severe hypoglycemia – Epidemiologic analysis of J-DOIT3 randomized controlled trial
Source: PLoS One. 2026 Oct 23;21(10):e0360295. doi: 10.1371/journal.pone.0360295 (PMC13693898; doi:10.1371/journal.pone.0360295)
Supplement: Supplementary file 5 [file pone.0360295.s005.docx]

**S5 Table.** Hazard ratios with stratified analyses by introducing categories to each variable

(a) Primary outcome (composed of occurrence of myocardial infarction, stroke, all-cause mortality, or revascularization)

|  |  |  |  |  | Men |  |  |  |
| --- | --- | --- | --- | --- | --- | --- | --- | --- |
| HbA1c (%) | Point estimate | 95%CI | P value |  | HDLC (mg/dL) | Point estimate | 95%CI | P value |
| 8.5 <= | 1 |  |  |  | <40 | 1 |  |  |
| 7.5 - <8.5 | 0.890 | 0.487 - 1.629 | 0.707 |  | 40 - <50 | 0.603 | 0.382 - 0.950 | **0.029** |
| 6.5 - <7.5 | 0.668 | 0.378 - 1.178 | 0.163 |  | 50 - <60 | 0.327 | 0.195 - 0.550 | **<0.001** |
| <6.5 | 0.463 | 0.239 - 0.897 | **0.022** |  | 60<= | 0.422 | 0.263 - 0.680 | **<0.001** |
|  |  |  |  |  | Women |  |  |  |
| SBP (mmHg) | Point estimate | 95%CI | P value |  | HDLC (mg/dL) | Point estimate | 95%CI | P value |
| 140<= | 1 |  |  |  | <40 | 1 |  |  |
| 130 - <140 | 0.840 | 0.533 - 1.324 | 0.454 |  | 40 - <50 | 0.590 | 0.181 - 1.919 | 0.380 |
| 120 - <130 | 0.623 | 0.397 - 0.978 | **0.040** |  | 50 - <60 | 0.425 | 0.133 - 1.355 | 0.148 |
| <120 | 0.753 | 0.478 - 1.185 | 0.220 |  | 60<= | 0.175 | 0.054 - 0.568 | **0.004** |
|  |  |  |  |  |  |  |  |  |
| LDLC (mg/dL) | Point estimate | 95%CI | P value |  |  |  |  |  |
| 120<= | 1 |  |  |  |  |  |  |  |
| 100 - <120 | 1.053 | 0.709 - 1.566 | 0.797 |  |  |  |  |  |
| 80 - <100 | 0.799 | 0.525 - 1.218 | 0.298 |  |  |  |  |  |
| <80 | 0.738 | 0.464 - 1.174 | 0.200 |  |  |  |  |  |

(b) Main secondary outcome (composite of myocardial infarction, stroke, and all-cause mortality)

|  |  |  |  |  | Men |  |  |  |
| --- | --- | --- | --- | --- | --- | --- | --- | --- |
| HbA1c (%) | Point estimate | 95%CI | P value |  | HDLC (mg/dL) | Point estimate | 95%CI | P value |
| 8.5 <= | 1 |  |  |  | <40 | 1 |  |  |
| 7.5 - <8.5 | 0.758 | 0.390 - 1.474 | 0.414 |  | 40 - <50 | 0.574 | 0.325 - 1.014 | 0.056 |
| 6.5 - <7.5 | 0.429 | 0.228 - 0.807 | **0.009** |  | 50 - <60 | 0.324 | 0.170 - 0.617 | **<.001** |
| <6.5 | 0.291 | 0.136 - 0.625 | **0.002** |  | 60<= | 0.453 | 0.253 - 0.810 | **0.008** |
|  |  |  |  |  | Women |  |  |  |
| SBP (mmHg) | Point estimate | 95%CI | P value |  | HDLC (mg/dL) | Point estimate | 95%CI | P value |
| 140<= | 1 |  |  |  | <40 | 1 |  |  |
| 130 - <140 | 0.675 | 0.384 - 1.185 | 0.171 |  | 40 - <50 | 0.533 | 0.149 - 1.905 | 0.333 |
| 120 - <130 | 0.567 | 0.330 - 0.975 | **0.040** |  | 50 - <60 | 0.234 | 0.064 - 0.861 | **0.029** |
| <120 | 0.740 | 0.430 - 1.273 | 0.276 |  | 60<= | 0.114 | 0.031 - 0.418 | **0.001** |
|  |  |  |  |  |  |  |  |  |
| LDLC (mg/dL) | Point estimate | 95%CI | P value |  |  |  |  |  |
| 120<= | 1 |  |  |  |  |  |  |  |
| 100 - <120 | 0.822 | 0.499 - 1.352 | 0.440 |  |  |  |  |  |
| 80 - <100 | 0.771 | 0.463 - 1.283 | 0.317 |  |  |  |  |  |
| <80 | 0.849 | 0.489 - 1.474 | 0.560 |  |  |  |  |  |

Hazard ratios for primary outcome (a) and main secondary outcome (b) were determined for each stratified variable by time-dependent Cox regression analysis, having the category of the lowest event rate as referent; for HDL-cholesterol, analysis was performed separately for men and women.

Bold indicates significant p-values.

NA: not available because of a small number of events.
