# Supplementary material for: Search for the glycated hemoglobin target minimizing mortality among people with type 2 diabetes with rare incidence of treatment-related severe hypoglycemia – Epidemiologic analysis of J-DOIT3 randomized controlled trial
Source: PLoS One. 2026 Oct 23;21(10):e0360295. doi: 10.1371/journal.pone.0360295 (PMC13693898; doi:10.1371/journal.pone.0360295)
Supplement: Supplementary file 6 [file pone.0360295.s006.docx]

**S6 Table.** Stratified analysis of the hazard ratios of HbA1c for all-cause and cancer mortality

(a) Stratified analysis of the hazard ratios of HbA1c (1%) for all-cause mortality by introducing categories into mean HDL-cholesterol values up to the third year of follow-up

|  | HDL-cholesterol category* | Point estimate | 95%CI | | | P value |
| --- | --- | --- | --- | --- | --- | --- |
| Total | Q1 | 0.952 | 0.563 | - | 1.611 | 0.855 |
|  | Q2 | 1.204 | 0.484 | - | 2.995 | 0.690 |
|  | Q3 | 3.591 | 1.784 | - | 7.227 | **<0.001** |
|  | Q4 | 2.251 | 1.359 | - | 3.728 | **0.002** |
| Men | Q1 | 0.948 | 0.507 | - | 1.775 | 0.868 |
|  | Q2 | 1.547 | 0.581 | - | 4.115 | 0.382 |
|  | Q3 | 4.784 | 1.612 | - | 14.197 | **0.005** |
|  | Q4 | 1.883 | 0.997 | - | 3.556 | 0.051 |
| Women | Q1 | 1.388 | 0.402 | - | 4.794 | 0.604 |
|  | Q2 | 0.396 | 0.028 | - | 5.662 | 0.495 |
|  | Q3 | 9.633 | 0.985 | - | 94.223 | 0.052 |
|  | Q4 | 2.654 | 0.994 | - | 7.088 | 0.051 |

(b) Stratified analysis of the hazard ratios of HbA1c (1%) for cancer mortality by introducing categories into mean HDL-cholesterol values up to the third year of follow-up, with other deaths being considered as competing risk

|  | HDL-cholesterol category* | Point estimate | 95%CI | | | P value |
| --- | --- | --- | --- | --- | --- | --- |
| Total | Q1 | 1.109 | 0.585 | - | 2.101 | 0.751 |
|  | Q2 | 0.969 | 0.314 | - | 2.988 | 0.956 |
|  | Q3 | 4.571 | 2.092 | - | 9.990 | **<0.001** |
|  | Q4 | 2.046 | 1.027 | - | 4.076 | **0.042** |
| Men | Q1 | 1.120 | 0.395 | - | 3.175 | 0.831 |
|  | Q2 | 1.125 | 0.327 | - | 3.865 | 0.852 |
|  | Q3 | 3.679 | 0.411 | - | 32.951 | 0.244 |
|  | Q4 | 1.630 | 0.831 | - | 3.199 | 0.155 |
| Women | Q1 | 1.388 | 0.337 | - | 5.726 | 0.650 |
|  | Q2 | 0.258 | 0.020 | - | 3.261 | 0.295 |
|  | Q3 | 4.163×10^8^ | 6.69×10^7^ | - | 2.59×10^9^ | **<0.001** |
|  | Q4 | 3.045 | 0.786 | - | 11.788 | 0.107 |

The HDL-cholesterol value was categorized into quartiles according to the mean HDL-cholesterol values in the participants up to the third year of follow-up. For cancer mortality, with other deaths being considered as a competing risk.

* HDL-cholesterol category Q1: ≤48.0, Q2: 48.0< ≤56.3, Q3: 56.3< ≤67.0, Q4: 67.0<

Bold indicates significant p-values.
