# Supplementary material for: Search for the glycated hemoglobin target minimizing mortality among people with type 2 diabetes with rare incidence of treatment-related severe hypoglycemia – Epidemiologic analysis of J-DOIT3 randomized controlled trial
Source: PLoS One. 2026 Oct 23;21(10):e0360295. doi: 10.1371/journal.pone.0360295 (PMC13693898; doi:10.1371/journal.pone.0360295)
Supplement: Supplementary file 7 [file pone.0360295.s007.docx]

**S7 Table.** Hazard ratios with stratified analyses by introducing categories to each variable among participants with no history of cardiovascular events—that is, the primary prevention cohort

(a) Myocardial infarction

|  |  |  |  |  | Men |  |  |  |
| --- | --- | --- | --- | --- | --- | --- | --- | --- |
| HbA1c (%) | Point estimate | 95%CI | P value |  | HDLC (mg/dL) | Point estimate | 95%CI | P value |
| 8.5 ≤ | 1 |  |  |  | <40 | 1 |  |  |
| 7.5 - <8.5 | 0.322 | 0.055 - 1.880 | 0.208 |  | 40 - <50 | 0.353 | 0.087 - 1.430 | 0.145 |
| 6.5 - <7.5 | 0.105 | 0.018 - 0.604 | **0.012** |  | 50 - <60 | 0.180 | 0.032 - 1.001 | 0.050 |
| <6.5 | 0.045 | 0.003 - 0.591 | **0.018** |  | 60≤ | 0.145 | 0.026 - 0.821 | **0.029** |
|  |  |  |  |  | Women |  |  |  |
| SBP (mmHg) | Point estimate | 95%CI | P value |  | HDLC (mg/dL) | Point estimate | 95%CI | P value |
| 140≤ | 1 |  |  |  | <40 | 1 |  |  |
| 130 - <140 | 2.454 | 0.269 - 22.390 | 0.426 |  | 40 - <50 | NA | NA | NA |
| 120 - <130 | 1.715 | 0.186 - 15.815 | 0.634 |  | 50 - <60 | NA | NA | NA |
| <120 | 1.326 | 0.129 - 13.593 | 0.812 |  | 60≤ | NA | NA | NA |
|  |  |  |  |  |  |  |  |  |
| LDLC (mg/dL) | Point estimate | 95%CI | P value |  |  |  |  |  |
| 120≤ | 1 |  |  |  |  |  |  |  |
| 100 - <120 | 2.574 | 0.500 - 13.236 | 0.258 |  |  |  |  |  |
| 80 - <100 | 0.437 | 0.038 - 4.985 | 0.505 |  |  |  |  |  |
| <80 | 2.182 | 0.315 - 15.106 | 0.429 |  |  |  |  |  |

(b) Stroke

|  |  |  |  |  | Men |  |  |  |
| --- | --- | --- | --- | --- | --- | --- | --- | --- |
| HbA1c (%) | Point estimate | 95%CI | p value |  | HDLC (mg/dL) | Point estimate | 95%CI | p value |
| 8.5 ≤ | 1 |  |  |  | <40 | 1 |  |  |
| 7.5 - <8.5 | 0.653 | 0.168 - 2.532 | 0.537 |  | 40 - <50 | 0.567 | 0.175 - 1.845 | 0.346 |
| 6.5 - <7.5 | 0.515 | 0.142 - 1.874 | 0.314 |  | 50 - <60 | 0.154 | 0.029 - 0.825 | **0.029** |
| <6.5 | 0.418 | 0.076 - 2.306 | 0.317 |  | 60≤ | 0.300 | 0.077 - 1.173 | 0.083 |
|  |  |  |  |  | Women |  |  |  |
| SBP (mmHg) | Point estimate | 95%CI | p value |  | HDLC (mg/dL) | Point estimate | 95%CI | p value |
| 140≤ | 1 |  |  |  | <40 | NA | NA | NA |
| 130 - <140 | 0.271 | 0.089 - 0.824 | **0.021** |  | 40 - <50 | 1 |  |  |
| 120 - <130 | 0.410 | 0.160 - 1.051 | 0.063 |  | 50 - <60 | 0.639 | 0.158 - 2.589 | 0.530 |
| <120 | 0.257 | 0.082 - 0.805 | **0.020** |  | 60≤ | 0.312 | 0.068 - 1.423 | 0.133 |
|  |  |  |  |  |  |  |  |  |
| LDLC (mg/dL) | Point estimate | 95%CI | p value |  |  |  |  |  |
| 120≤ | 1 |  |  |  |  |  |  |  |
| 100 - <120 | 0.577 | 0.243 - 1.372 | 0.214 |  |  |  |  |  |
| 80 - <100 | 0.332 | 0.114 - 0.968 | **0.043** |  |  |  |  |  |
| <80 | 0.178 | 0.036 - 0.887 | **0.035** |  |  |  |  |  |

(c) All-cause mortality

|  |  |  |  |  | Men |  |  |  |
| --- | --- | --- | --- | --- | --- | --- | --- | --- |
| HbA1c (%) | Point estimate | 95%CI | P value |  | HDLC (mg/dL) | Point estimate | 95%CI | P value |
| 8.5 ≤ | 1 |  |  |  | <40 | 1 |  |  |
| 7.5 - <8.5 | 1.357 | 0.394 - 4.677 | 0.628 |  | 40 - <50 | 0.595 | 0.236 - 1.497 | 0.270 |
| 6.5 - <7.5 | 0.722 | 0.217 - 2.409 | 0.597 |  | 50 - <60 | 0.386 | 0.140 - 1.061 | 0.065 |
| <6.5 | 0.358 | 0.090 - 1.428 | 0.146 |  | 60≤ | 0.589 | 0.245 - 1.419 | 0.238 |
|  |  |  |  |  | Women |  |  |  |
| SBP (mmHg) | Point estimate | 95%CI | P value |  | HDLC (mg/dL) | Point estimate | 95%CI | P value |
| 140≤ | 1 |  |  |  | <40 | 1 |  |  |
| 130 - <140 | 1.100 | 0.425 - 2.847 | 0.844 |  | 40 - <50 | 0.251 | 0.057 - 1.105 | 0.068 |
| 120 - <130 | 0.816 | 0.320 - 2.081 | 0.671 |  | 50 - <60 | 0.125 | 0.029 - 0.541 | **0.005** |
| <120 | 0.919 | 0.354 - 2.382 | 0.861 |  | 60≤ | 0.060 | 0.013 - 0.284 | **<0.001** |
|  |  |  |  |  |  |  |  |  |
| LDLC (mg/dL) | Point estimate | 95%CI | P value |  |  |  |  |  |
| 120≤ | 1 |  |  |  |  |  |  |  |
| 100 - <120 | 1.044 | 0.418 - 2.609 | 0.926 |  |  |  |  |  |
| 80 - <100 | 2.018 | 0.880 - 4.626 | 0.097 |  |  |  |  |  |
| <80 | 2.089 | 0.844 - 5.167 | 0.111 |  |  |  |  |  |

Hazard ratios for myocardial infarction (a), stroke (b), and all- cause mortality (c) were determined for each stratified variable by time-dependent Cox regression analysis, having the category of the lowest event rate as referent; for HDL-cholesterol, analysis was performed separately for men and women.

Bold indicates significant p-values.

NA: not available because of a small number of events. (a) There were no cases of myocardial infarction among women. (b) Since no cases of stroke were observed in women with HDL-C levels <40 mg/dL, this category is designated as NA.
