# Supplementary material for: Search for the glycated hemoglobin target minimizing mortality among people with type 2 diabetes with rare incidence of treatment-related severe hypoglycemia – Epidemiologic analysis of J-DOIT3 randomized controlled trial
Source: PLoS One. 2026 Oct 23;21(10):e0360295. doi: 10.1371/journal.pone.0360295 (PMC13693898; doi:10.1371/journal.pone.0360295)
Supplement: Supplementary file 8 [file pone.0360295.s008.docx]

**S8 Table.** Cause of death

| **Cause -- no. (%)** | **Conventional**  **(N = 1247)** | **Intensive**  **(N = 1239)** |
| --- | --- | --- |
| Malignant neoplasms | 24 (1.9) | 32 (2.6) |
| Myocardial infarction | 0 (0.0) | 0 (0.0) |
| Stroke | 3 (0.2) | 0 (0.0) |
| Heart failure | 1 (0.1) | 0 (0.0) |
| Liver failure | 1 (0.1) | 4 (0.3) |
| Pneumonia | 3 (0.2) | 0 (0.0) |
| Other infectious diseases | 2 (0.2) | 0 (0.0) |
| Interstitial pneumonia | 4 (0.3) | 0 (0.0) |
| Sudden death | 2 (0.2) | 2 (0.2) |
| Accident | 1 (0.1) | 0 (0.0) |
| Suicide | 0 (0.0) | 2 (0.2) |
| Others | 2 (0.2) | 1 (0.1) |
| Undetermined | 3 (0.2) | 8 (0.6) |
| All causes | 46 (3.7) | 49 (4.0) |

The number of deaths for each cause during the term after the landmark time-point is indicated. Figures in parentheses indicate percentages of the total population of each intervention group.
