# Supplementary material for: Search for the glycated hemoglobin target minimizing mortality among people with type 2 diabetes with rare incidence of treatment-related severe hypoglycemia – Epidemiologic analysis of J-DOIT3 randomized controlled trial
Source: PLoS One. 2026 Oct 23;21(10):e0360295. doi: 10.1371/journal.pone.0360295 (PMC13693898; doi:10.1371/journal.pone.0360295)
Supplement: Supplementary file 9 [file pone.0360295.s009.docx]

**Appendix**

Inclusion criteria

Patients were considered eligible for inclusion if they were 45 years old or older but younger than 70 years old at study entry, had type 2 diabetes, and met both “(1) and (2)” or both “(1) and (3)” described below. Those who met all three “(1), (2) and (3)” were also considered eligible for inclusion.

(1) Glycemic control

Those with HbA1c 6.9% or greater despite treatment with any of the three regimens given below.

1) Diet and exercise therapy alone

2) Diet and exercise therapy plus 1 oral anti-diabetic drug

3) Diet and exercise therapy plus αGI and 1 other oral anti-diabetic drug

(2) Blood pressure control

Those with the following casual blood pressure (BP) level, as measured on an outpatient basis

1) Systolic BP ≥ 140 mmHg or diastolic BP ≥ 90 mmHg while not on an antihypertensive agent

2) Systolic BP ≥ 130 mmHg or diastolic BP ≥ 80 mmHg while on 1 or 2 ARB, ACEI, or long-acting CCB

Those receiving antihypertensive agents other than ARB, ACEI, or long-acting CCB were not eligible for study entry, with the exception of those who were receiving these agents for other purposes than blood pressure lowering.

(3) Lipid control

Those with the following fasting lipid levels while not on a lipid-lowering agent

1) LDL-cholesterol, ≥ 120 mg/dL (as estimated by using the Friedewald formula)

2) Triglycerides, ≥ 150 mg/dL

3) HDL-cholesterol, < 40 mg/dL

Patients receiving 1 lipid-lowering agent were judged eligible for study entry if they met any of the above criteria. However, those on fibrates were to discontinue that fibrate treatment at the start of the study when they were assigned to the intensive therapy arm.

Exclusion criteria

1. Those with poorly controlled hypertension despite pharmacological therapy (systolic BP ≥ 200 mmHg or diastolic BP ≥ 120 mmHg)

2. Those on insulin therapy

3. Those with non-diabetic renal disease

4. Those in whom type 1 and other diabetes due to pathogenic mechanisms other than those associated with type 2 diabetes were strongly suspected

5. Those who tested anti-GAD antibody-positive

6. Those with LDL cholesterol ≥ 200 mg/dL

7. Those suspected of having secondary hypertension other than renal parenchymal hypertension

8. Those suspected of having a hereditary lipid disorder with a strong family history of lipid metabolic disorder

9. Those who were receiving antihypertensive agents other than ARB, ACEI, long-acting CCB, except where they were receiving these agents for other purposes than blood pressure lowering

10. Those who were receiving 3 or more antihypertensive agents (i.e., ARB, ACEI, and long-acting CCB), except where they were receiving these agents for other purposes than blood pressure lowering

11. Those with more serious retinopathy than proliferative retinopathy

12. Renal failure (serum Cr: ≥ 2.0 mg/dL in men; ≥ 1.5 mg/dL in women)

13. Those with a history of cardiac failure or those with cardiac failure

14. Those who were pregnant or potentially pregnant

15. Those who met any of the following criteria and who had BNP ≥ 100 pg/mL

⚫ Myocardial infarction

⚫ Angina pectoris (or a history of disease)

⚫ History of coronary artery bypass graft (CABG)

⚫ History of percutaneous coronary angioplasty (PTCA)

⚫ Other cardiac disease

⚫ ECG findings of left ventricular hyperplasia

⚫ Abnormal ECG findings (excluding isolated extrasystole or right bundle branch block [RBBB])

16. Those judged by the attending physician to be ineligible for study entry

ARB: angiotensin II receptor blocker, ACEI: angiotensin converting enzyme inhibitor, CCB: calcium channel blocker.
