# Supplementary material for: Search for the glycated hemoglobin target minimizing mortality among people with type 2 diabetes with rare incidence of treatment-related severe hypoglycemia – Epidemiologic analysis of J-DOIT3 randomized controlled trial
Source: PLoS One. 2026 Oct 23;21(10):e0360295. doi: 10.1371/journal.pone.0360295 (PMC13693898; doi:10.1371/journal.pone.0360295)
Supplement: Supplementary file 11 [file pone.0360295.s013.docx]

The following are the authors’ responses to the comments from the editor and reviewers.

From and To the Editor

1. The small number of outcome events, particularly myocardial infarction (n = 16), represents a critical limitation. The stratified threshold analyses presented in Table 4, which underpin your central recommendation of an HbA1c target of <6.5%, are considered statistically weak. You are required to reframe non-significant findings with appropriate caution, explicitly acknowledging the inability to distinguish true null effects from type II errors. Furthermore, instances yielding "NA" results due to insufficient events must be accompanied by discussion rather than left without comment.

Response:

We appreciate your thoughtful feedback. Based on your comments, we have made the following revisions to tone down the wording. We deleted “Rather, it appeared to increase from the lowest to the highest HbA1c category, especially for all-cause mortality and myocardial infarction, suggesting the absence of any J curve-like association. (lines 407-409; in a marked-up copy hereafter)” Instead, we added the following. “However, we should be very cautious, especially regarding myocardial infarction, because statistically significant differences in the second and third categories might have been overlooked due to the small number of events. (lines 410-412).”

In addition, we have also revised the relevant sections in the "Conclusions" of the ABSTRACT, and in the second and final paragraphs of the "DISCUSSION" section.

We also added a limitation stating that among women, we were unable to fully perform an analysis of the association between HDL-cholesterol and myocardial infarction, as well as a part of stroke, due to the small number of events (lines 469-471), and provided the specific numbers of events as a footnote of this table.

2. The 1-year landmark design introduces a potential healthy survivor bias, as participants experiencing early events or discontinuing treatment prior to the landmark were excluded. The exclusion of 75 participants due to missing variables also requires explicit justification. Please provide relevant sensitivity analyses and a thorough discussion of how these exclusions may have affected the hazard ratio estimates.

Response:

We conducted an analysis using updated mean values in order to assess the direct effects of treatment during the intervention period. Furthermore, to minimize the influence of pre-registration treatment as much as possible, we attempted landmark analysis with a landmark time of 1-year that we assumed to be adequate and employed this term. Given the nature of this landmark analysis, which requires values from the first term, we performed a sensitivity analysis using a 6-month (180-day) landmark time-point (with 2507 participants) instead of 1 year. However, although there was one death in the latter half of the first year, the data for the variables of this participant were missing, and it was not included in the analysis; there were no cases of myocardial infarction during this period. Therefore, the results remained virtually unchanged (the changes in those values were by far smaller than the smallest digit in the table) for both of these two outcomes. With regard to stroke, seven cases with no missing data were identified during this period; the results are presented in Supplemental Table 4 (re-numbered). Alteration of the landmark time-point did not show a substantial difference in the stroke, either. We have added this information to the RESULTS section (lines 352-360).

In addition, regarding the 75 participants excluded due to missing data for variables when using the landmark time-point of 1 year, we have specified the details and included this information in the RESULTS section (lines 290-293).

3. The statement that there is "no lower threshold for HbA1c," and the resulting recommendation of a <6.5% target, is not adequately supported by the data. Given that the <6.5% category was not further subdivided and that confidence intervals are extremely wide, the absolute lower limit of safe glycemic control cannot be established from this dataset alone. The authors are advised to qualify this conclusion.

Response:

We have deleted the part of “no lower threshold for HbA1c”. Instead, we added a phrase “although given that the category of <6.5% has not been subdivided and the confidence interval is wide, our analysis does not identify the absolute lower limit for safe blood glucose control.” (lines 461-463)

4. The manuscript does not sufficiently address the possibility that observed outcome improvements reflect the pleiotropic cardiovascular effects of specific drug classes, such as SGLT2 inhibitors and GLP-1 receptor agonists, rather than HbA1c reduction per se. Additionally, the omission of renal outcomes, BMI, and triglyceride levels from the analyses requires justification, as these represent clinically relevant covariates whose absence limits the completeness of the risk factor evaluation.

Response:

This study was completed in March 2016. At that time-point, below 5% of patients had been prescribed GLP-1 receptor agonists at the final visit in both the intensive and conventional therapy groups, which was also the case with SGLT2 inhibitors, as shown in the Supplementary table 2 of the ref. #8 of the manuscript; therefore, it is unlikely that this had a significant impact on the results. We added this sentence to the DISCUSSION section. (lines 482-487)

Renal outcomes have been separated out as microvascular events and have been discussed in detail in a separate paper already published (Kidney Int. 2021 Jan;99(1):256-266).

The purpose of the current analysis was to identify factors mediating the effects of intervention of the J-DOIT3; because HDL cholesterol levels showed a significant difference between the intensive and conventional therapy groups, as shown in Figure 2 of the main analysis paper (ref. #8 of the manuscript), this variable was included in the analysis. Since there was only a small difference in BMI trends between the two groups, as shown in the same figure, it was not included as an analytical variable. Regarding triglycerides, since we included HDL-cholesterol as described above, triglycerides were omitted, considering the correlation between the two. This was also the case with the procedure that only systolic blood pressure was employed, rather than both systolic and diastolic blood pressure. These sentences are now included (below Table 3).

From and To the Reviewer #1:

I am pleased to have the opportunity to review your manuscript, 'PONE-D-26-09852'. Your study addresses a critical gap in diabetes management by re-evaluating HbA1c targets in a contemporary clinical environment in which severe hypoglycaemia is uncommon. The use of updated mean values and the landmark analysis approach are both statistically sound, providing a dynamic view of risk factor management.

However, to strengthen the clinical applicability and robustness of your findings further, I have the following comments and requests for additional analysis:

Response:

We greatly appreciate your encouraging comment above.

Regarding the points you raised, our response is as follows.

1. The study population predominantly consists of participants for primary prevention, with only 11.4% having a history of cardiovascular disease. Although baseline cardiovascular disease (CVD) history was used as a covariate in your Cox regression models, the risk structures for primary and secondary prevention are fundamentally different. Given that primary prevention represents the vast majority of your cohort, please perform a sensitivity analysis extracting only the primary prevention group and confirm that the tendency for all-cause mortality and myocardial infarction remains consistent within this subgroup as it does in the total population.

Response:

Thank you very much for your thoughtful suggestion. We have performed your proposal as a sensitivity analysis. The result is now included in Supplemental Table 7, and the tendency for all-cause mortality and myocardial infarction remains basically consistent within this sub-cohort to that in the total population. We have added this information in the last part of the RESULTS section (lines 372-376).

2. Your analysis begins at the landmark time point of 1 year after baseline. This approach may introduce a 'healthy survivor bias', as participants who experienced events or discontinued treatment early on are excluded. Please discuss how the exclusion of these early-phase cases might have influenced the observed hazard ratios.

Response:

Thank you very much for your important comment. Regarding this point, please refer to our responses to the Editor’s Comment 2.

3. The J-DOIT3 study period coincided with an increased use of newer agents, such as SGLT2 inhibitors and GLP-1 receptor agonists. It is unclear whether improved outcomes are solely attributable to the numerical reduction in HbA1c, or whether the pleiotropic effects of specific drug classes also played a role. Please address the potential confounding effect of medication types on your results.

Response:

Thank you very much for your valuable feedback. On this point, please refer to our response to the Editor’s comment 4.

4. The conclusion states that there is no clear lower threshold for HbA1c based on the lowest category of <6.5%. As this lowest category was not further subdivided, the assertion that 'no lower threshold exists' should be carefully qualified. Please discuss the limitations of the data in defining an absolute lower limit.

Response:

We appreciate your pointing out this. Regarding this issue, please refer to our response to the Editor’s comment 3.

5. HDL-C had a notable impact on different outcomes in men and women.

Please provide a more detailed biological or clinical explanation as to why the protective role of HDL-C appears to be sex-specific in your cohort.

Thank you for your insightful comment. Inspired by your suggestion, we have incorporated the following points into the discussion (lines 447-451) and added two papers to reference (re-numbered #29 & #30).

“Although it is difficult to draw a precise picture regarding gender differences in the correlation between stroke and HDL cholesterol, and between all-cause mortality and HDL cholesterol, our analysis may be in line with the findings that, in the general Japanese population, the correlation with HDL cholesterol tends to be stronger for all-cause mortality among women, and for stroke among men.”

From and To the Reviewer #2:

The team intended to identify the thresholds of HbA1c and other clinical determinants associated with a minimized risk of macrovascular complications of diabetes in the setting of the J-DOIT3 trial.

Response:

We greatly appreciate your insightful expression above.

1. How the participants were assigned need clarification. For example, how to implement based on age (exact match?) and so on?

Response:

We are very grateful if you could kindly refer to the design paper (ref. #9 of the manuscript) as this information is included therein.

2. The measures after one year landmark time point were collected which implies the exclusion of early outcome estimation. Is there any early treatment effects or risk?

Response:

Thank you very much for your valuable comment. Regarding this point, please refer to our responses to the Editor’s Comment 2.

3. Cox regression was implemented. Is the proportional hazard assumption validated?

Response:

The proportional hazards assumption for the treatment group was assessed by visual inspection of log-minus-log survival plots. We acknowledge that, in randomized controlled trials, treatment effects may vary over time and strict proportionality may not always hold throughout the entire follow-up period.

To address this issue, we additionally evaluated models incorporating time-dependent covariates and treatment-by-covariate interaction terms in this report. The inclusion of time-dependent covariates and interaction terms was intended to account for potential non-proportionality and to evaluate the robustness of the findings under less restrictive modeling assumptions. These analyses yielded results that were materially unchanged from the primary analysis of J-DOIT3, supporting the robustness of the reported treatment effect.

4. While the sample size is large, some of events are small, e.g., n= 16 for MI. Please comment on the robustness of the reported results and the power for the analysis.

Response:

We appreciate your important comments. We have toned down our claims regarding the result of myocardial infarction, given the small number of events. Please refer to our responses to the Editor’s Comment 1 regarding this point. We have also conducted sensitivity analyses to assess the robustness of the results, one as mentioned above (Response #2), and we also extracted and analyzed only participants with no history of cardiovascular events—that is, the primary prevention cohort (Supplementary Table 7) as another sensitivity analysis.

5. 75 participants were excluded from the analysis. Please comment on whether these may lead to any biased results.

Response:

As you pointed out, we excluded 75 participants due to insufficient data for adjustment. Regarding these 75 participants, we newly described the specific breakdown is in lines 290-293. Because of this, we are not able to rule out the possibility that this might have introduced some bias. This point is now mentioned as the fourth limitation (lines 473- 475).

6. Threshold analysis with stratified analysis in Table 4 is concerning, especially with small number of events. Also, comment on NA results!

Also, please comment on how these thresholds were determined

Response:

We appreciate your comments very much. We have toned down our claims regarding the result of myocardial infarction, given the small number of events. Regarding this point, please refer to our responses to the Editor’s Comment 1.

We also added a limitation stating that among women, we were unable to fully perform an analysis of the association between HDL-cholesterol and myocardial infarction, as well as a part of stroke, due to the small number of events (lines 469-471), and provided the specific numbers of events as a footnote of Table 4.

7. Interpretation on the not significant results should be more cautious as the power may be limited for some of these analysis

Response:

Thank you for your important comment. With regard to this point, we have made the following revisions to tone down the wording. We deleted a sentence (lines 407-409) and, instead, we have added the following. “However, we should be very cautious, especially regarding myocardial infarction, because statistically significant differences in the second and third categories might have been overlooked due to the small number of events. (lines 410-412).”

From and To the Reviewer #3:

The manuscript is well written, and the findings are clearly presented with adequate supporting data. The paper is well organized and written in standard English. Overall, the study is methodologically sound, and I recommend it for publication.

Response:

Thank you very much for your kind comments on the significance, content, and methodology of my paper. We are deeply grateful for your recommendation for publication.

From and To the Reviewer #4:

This study is a sub-analysis to verify the optimal management target values for each individual risk factor in the J-DOIT3 study. The results obtained are very interesting, but the authors need to reconsider the following points.

Response:

Thank you for your feedback and advice. Based on your comments, we have revised the manuscript as follows.

1. The J-DOIT3 study also evaluated renal outcomes, but why were they not included in this analysis?

Response:

Renal outcomes have been separated out as microvascular events and have been discussed in detail in a separate paper already published (Kidney Int. 2021 Jan;99(1):256-266).

2. To improve the visual clarity of research results and enhance reader comprehension, forest plots are preferable to tables.

Response:

As is common in epidemiological studies, we used tables rather than forest plots. We believe that tables provide information essential to scientific evidence, such as “detailed data disclosure,” “comprehensive comparative analysis,” and “presentation of results adjusted for confounding factors.”

3. What are the optimal values for BMI and triglycerides to prevent cardiovascular events?

Response:

The purpose of the current analysis was to identify factors mediating the effects of intervention of the J-DOIT3; because HDL cholesterol levels showed a significant difference between the intensive and conventional therapy groups, as shown in Figure 2 of the main analysis paper (ref. #8 of the manuscript), this variable was included in the analysis. Since there was only a small difference in BMI trends between the two groups, as shown in the same figure, it was not included as an analytical variable. Regarding triglycerides, since we included HDL-cholesterol as described above, triglycerides were omitted, considering the correlation between the two. This was also the case with the procedure that only systolic blood pressure was employed, rather than both systolic and diastolic blood pressure. These sentences are now included (below Table 3).

4. The abstract must be no more than 300 words, so it needs to be kept short to comply with the requirements.

Response:

According to your advice, we have deleted more than one phrase of a sentence of the abstract, and now the word count is 299 except section titles and fundings.

From and To the Reviewer #5:

Dear Authors,

This manuscript addresses an important subject by considering the use of new drugs for diabetes treatment associated with fewer episodes of severe hypoglycemia and the incidence of macrovascular complications.

Despite the limitations of an open-label study, it provides data that would help establish target levels of HbA1C, SBP and LDL-c to prevent macrovascular complications. However, it is necessary to make some revisions and clarifications described below.

Response:

We greatly appreciate your comments, right to the point, and we have amended the manuscript according to your queries.

#1 It is necessary to make grammatical and punctuation corrections in the whole manuscript.

Response:

Thank you very much. Based on your advice, we have reviewed and amended the grammatical errors throughout the entire text.

#2 The methods deserve clarification.

- The study describes 2 groups of participants with different treatment targets, and Table 1 shows baseline data for a landmark point, which appears to be the data reported when all participants were already included in the study, but it is not very clear.

Response:

Table 1 presents data for all participants one year after enrollment. To clarify this point, a new sentence has been added at the beginning of the footnote.

- It also says that participants' data from the year after that landmark point would be in the supplementary material (Table 2). Supplementary Table 2 shows the combined data for the 2 groups, and the mean levels of HbA1C, LDL, and SBP are outside the pre-established targets for both groups. It is important to report the data for each group separately, as well as the distributions of the number of patients who achieved, stratified by the analysis stratification shown in the results.

Response:

The trends in the data for each group are presented in the main analysis paper (ref. #8); in this table, we intended to present the data used for the combined landmark analysis of both groups.

#3 It is worth including in the discussion comments on the real differences or similarities between the 2 groups and the combined analysis, which may affect the conclusions.

Response:

Although the policy to combine the two groups for analysis is noted at the beginning of the Results section, we also reiterated this point in the first paragraph of the DISCUSSION section (lines 400-404).

Other than the above-mentioned, we have adjusted the tables’ formatting, but the content has not been changed. Also, the name of the institution where one of the authors is affiliated has been changed, so this has been updated accordingly. Also, affiliation change for a new physical year in Japan We have also updated the information to reflect changes in the affiliated institution that commenced with the new fiscal year in Japan beginning in April.
