# Supplementary material for: Search for the glycated hemoglobin target minimizing mortality among people with type 2 diabetes with rare incidence of treatment-related severe hypoglycemia – Epidemiologic analysis of J-DOIT3 randomized controlled trial
Source: PLoS One. 2026 Oct 23;21(10):e0360295. doi: 10.1371/journal.pone.0360295 (PMC13693898; doi:10.1371/journal.pone.0360295)
Supplement: Supplementary file 12 [file pone.0360295.s014.docx]

The following are the authors’ responses to the comments from the editor and reviewers.

From and To the Reviewer #4:

Although there are some differences of opinion between the authors and the reviewer, I would like to comment on the following points.

Response:

Thank you for your important suggestions. Based on your comments, we have revised the manuscript as follows.

1. The authors have stated that they have already published findings on the renal outcomes of the J-DOIT3 study (Kidney Int. 2021. Jan;99(1):256-266), but this should be mentioned in the text and included in the references. It should be noted that not all readers are familiar with the J-DOIT3 study.

Response:

Thank you very much for your constructive and receptive comment. We have added the following text as the final paragraph of the “Introduction” section (lines 131-133), citing the relevant reference (#16) that you mentioned.

“It should be noted here that detailed results and discussions regarding renal outcomes—which are no less important than cardiovascular outcomes in diabetic vascular complications—have already been reported in our previous publication.^16^”

2. Forest plots can adequately represent the adjustment for confounding factors and provide sufficient scientific evidence. My opinion remains that they are a useful method for busy readers to quickly and visually grasp the outline of the research, but I respect the authors' decision to present the information in a table.

Response:

Thank you for your valuable insights and for accepting our approach on this matter. We think each of these approaches has its own advantages, and we imagine you would probably share this view.

From and To the Reviewer #5:

I am glad to rereview this manuscript. The authors addressed most of the reviewers' comments and added some important observations.

Response:

Thank you very much for your kind words. We, the authors, are truly encouraged by your positive feedback.

However, considering the data from your previous study, only part of the participants in the study achieved the pre-established goals of treatment in both groups, especially in the intensive treatment group where only 18% achived HbA1C below 6.2%, with a mean level of 6,8%( reference data) ; it is still considered important to make this clear in the discussion, as the low incidence of severe hypoglycemia may be affected by such data, and strict control could possibly affect the number of events and the significance of the results related to the safety of lower levels of HbA1C.

Response:

We appreciate your more detailed and specific important comment. In response to your comment, we have amended the manuscript by adding a cohesive pair of sentences as the fifth limitation as follows (lines 463-468). Moreover, in connection with this, we have toned down the nuance of the final part of the “discussion” section (lines 481-482).

“Fifth, in the J-DOIT3 study, only a part of participants in both groups achieved the pre-specified HbA1c treatment targets; in particular, in the intensive therapy group, the mean HbA1c during the intervention period was 6.8%, and only 18% achieved an HbA1c level below the target of 6.2%. This may have masked the occurrence of events at further lower HbA1c levels, potentially resulting in insufficient evaluation of HbA1c at those levels. It is crucial to note that this analysis does not guarantee safety at very low HbA1c levels.”
