# Supplementary material for: Search for the glycated hemoglobin target minimizing mortality among people with type 2 diabetes with rare incidence of treatment-related severe hypoglycemia – Epidemiologic analysis of J-DOIT3 randomized controlled trial
Source: PLoS One. 2026 Oct 23;21(10):e0360295. doi: 10.1371/journal.pone.0360295 (PMC13693898; doi:10.1371/journal.pone.0360295)
Supplement: Peer review history

# Peer review: Decision Letter 0

**DOI:** 10.1371/journal.pone.0360295.r001

**Authors:** Hidetaka Hamasaki

22 Apr 2026

Dear Dr. Sasako,

**ACADEMIC EDITOR:**

Dear Authors,

Thank you for submitting your valuable work to PLOS ONE.

After careful evaluation by multiple reviewers, we have identified several concerns that must be addressed before we can consider your manuscript for publication. As an Academic Editor, I am particularly concerned about the following points and respectfully request that you revise your manuscript accordingly.

The small number of outcome events, particularly myocardial infarction (n = 16), represents a critical limitation. The stratified threshold analyses presented in Table 4, which underpin your central recommendation of an HbA1c target of <6.5%, are considered statistically weak. You are required to reframe non-significant findings with appropriate caution, explicitly acknowledging the inability to distinguish true null effects from type II errors. Furthermore, instances yielding "NA" results due to insufficient events must be accompanied by discussion rather than left without comment.

The 1-year landmark design introduces a potential healthy survivor bias, as participants experiencing early events or discontinuing treatment prior to the landmark were excluded. The exclusion of 75 participants due to missing variables also requires explicit justification. Please provide relevant sensitivity analyses and a thorough discussion of how these exclusions may have affected the hazard ratio estimates.

The statement that there is "no lower threshold for HbA1c," and the resulting recommendation of a <6.5% target, is not adequately supported by the data. Given that the <6.5% category was not further subdivided and that confidence intervals are extremely wide, the absolute lower limit of safe glycemic control cannot be established from this dataset alone. The authors are advised to qualify this conclusion.

The manuscript does not sufficiently address the possibility that observed outcome improvements reflect the pleiotropic cardiovascular effects of specific drug classes, such as SGLT2 inhibitors and GLP-1 receptor agonists, rather than HbA1c reduction per se. Additionally, the omission of renal outcomes, BMI, and triglyceride levels from the analyses requires justification, as these represent clinically relevant covariates whose absence limits the completeness of the risk factor evaluation.

While the reviewers recognize the clinical importance of the research question and acknowledge genuine strengths in the study design, the manuscript requires major revision. We look forward to receiving your revised manuscript.

==============================

Please submit your revised manuscript by Jun 06 2026 11:59PM. If you will need more time than this to complete your revisions, please reply to this message or contact the journal office at plosone@plos.org. When you're ready to submit your revision, log on to https://www.editorialmanager.com/pone/ and select the 'Submissions Needing Revision' folder to locate your manuscript file.

Please include the following items when submitting your revised manuscript:

- A letter that responds to each point raised by the academic editor and reviewer(s). You should upload this letter as a separate file labeled 'Response to Reviewers'.
- A marked-up copy of your manuscript that highlights changes made to the original version. You should upload this as a separate file labeled 'Revised Manuscript with Track Changes'.
- An unmarked version of your revised paper without tracked changes. You should upload this as a separate file labeled 'Manuscript'.

If you would like to make changes to your financial disclosure, please include your updated statement in your cover letter. Guidelines for resubmitting your figure files are available below the reviewer comments at the end of this letter.

If applicable, we recommend that you deposit your laboratory protocols in protocols.io to enhance the reproducibility of your results. Protocols.io assigns your protocol its own identifier (DOI) so that it can be cited independently in the future. For instructions see: https://journals.plos.org/plosone/s/submission-guidelines#loc-laboratory-protocols. Additionally, PLOS ONE offers an option for publishing peer-reviewed Lab Protocol articles, which describe protocols hosted on protocols.io. Read more information on sharing protocols at https://plos.org/protocols?utm_medium=editorial-email&utm_source=authorletters&utm_campaign=protocols.

As the corresponding author, your ORCID iD is verified in the submission system and will appear in the published article. PLOS supports the use of ORCID, and we encourage all coauthors to register for an ORCID iD and use it as well. Please encourage your coauthors to verify their ORCID iD within the submission system before final acceptance, as unverified ORCID iDs will not appear in the published article. *Only*  the individual author can complete the verification step; PLOS staff *cannot*  verify ORCID iDs on behalf of authors.

We look forward to receiving your revised manuscript.

Kind regards,

Hidetaka Hamasaki

Academic Editor

PLOS One

Journal Requirements:

When submitting your revision, we need you to address these additional requirements.

1. Please ensure that your manuscript meets PLOS ONE's style requirements, including those for file naming. The PLOS ONE style templates can be found at

https://journals.plos.org/plosone/s/file?id=wjVg/PLOSOne_formatting_sample_main_body.pdf and

https://journals.plos.org/plosone/s/file?id=ba62/PLOSOne_formatting_sample_title_authors_affiliations.pdf

2. Thank you for stating the following in the Acknowledgments Section of your manuscript:

"The authors are very grateful to Prof. Atsushi Goto of Yokohama City University for his helpful discussions.

Financial Support: This study was supported by the Ministry of Health, Labour and Welfare of Japan, the Japan Agency for Medical Research and Development, the Japan Diabetes Society, the Manpei Suzuki Diabetes Foundation, Asahi Kasei Pharma, Astellas Pharma, AstraZeneca, Bayer, Boehringer Ingelheim, Bristol-Myers Squibb, Daiichi Sankyo, Eli Lilly, GlaxoSmithKline, Kissei Pharmaceutical, Kowa, Mitsubishi Tanabe Pharma, Mochida Pharmaceutical, MSD, Novartis Pharma, Novo Nordisk, Ono Pharmaceutical, Pfizer, Sanwa Kagaku Kenkyusho, Shionogi, Sumitomo Dainippon Pharma, Taisho Pharmaceutical, and Takeda."

We note that you have provided additional information within the Acknowledgements Section that is not currently declared in your Funding Statement. Please note that funding information should not appear in the Acknowledgments section or other areas of your manuscript. We will only publish funding information present in the Funding Statement section of the online submission form.

Please remove any funding-related text from the manuscript and let us know how you would like to update your Funding Statement. Currently, your Funding Statement reads as follows:

"Ministry of Health, Labour and Welfare of Japan, Japan Agency for Medical Research and Development, Japan Diabetes Society, Manpei Suzuki Diabetes Foundation, Asahi Kasei Pharma, Astellas Pharma, AstraZeneca, Bayer, Boehringer Ingelheim, Bristol-Myers Squibb, Daiichi Sankyo, Eli Lilly, GlaxoSmithKline, Kissei Pharmaceutical, Kowa Pharmaceutical, Mitsubishi Tanabe Pharma, Mochida Pharmaceutical, MSD, Novartis Pharma, Novo Nordisk, Ono Pharmaceutical, Pfizer, Sanwa Kagaku Kenkyusho, Shionogi, Sumitomo Dainippon Pharma, Taisho Toyama Pharmaceutical, and Takeda."

Please include your amended statements within your cover letter; we will change the online submission form on your behalf.

3. Thank you for stating the following financial disclosure:

"Ministry of Health, Labour and Welfare of Japan, Japan Agency for Medical Research and Development, Japan Diabetes Society, Manpei Suzuki Diabetes Foundation, Asahi Kasei Pharma, Astellas Pharma, AstraZeneca, Bayer, Boehringer Ingelheim, Bristol-Myers Squibb, Daiichi Sankyo, Eli Lilly, GlaxoSmithKline, Kissei Pharmaceutical, Kowa Pharmaceutical, Mitsubishi Tanabe Pharma, Mochida Pharmaceutical, MSD, Novartis Pharma, Novo Nordisk, Ono Pharmaceutical, Pfizer, Sanwa Kagaku Kenkyusho, Shionogi, Sumitomo Dainippon Pharma, Taisho Toyama Pharmaceutical, and Takeda."

Please state what role the funders took in the study.  If the funders had no role, please state: "The funders had no role in study design, data collection and analysis, decision to publish, or preparation of the manuscript."

If this statement is not correct you must amend it as needed.

Please include this amended Role of Funder statement in your cover letter; we will change the online submission form on your behalf.

4. Thank you for stating the following in the Competing Interests section:

" KU reports grants and endowments from Astellas, Boehringer Ingelheim, Eli Lilly, Kyowa Kirin, Mitsubishi Tanabe, MSD, Novartis, Novo Nordisk, Ono, and Sumitomo; personal fees from AstraZeneca, Boehringer Ingelheim, Kyowa Kirin, Mitsubishi Tanabe, MSD, Novo Nordisk, Ono, Sanofi, Sumitomo, and Taisho; chairmanship of the Japan Diabetes Society. TS reports an endowment from Eli Lilly; personal fees from AstraZeneca, Boehringer Ingelheim, Daiichi Sankyo, Eli Lilly, Kowa, Novo Nordisk, Ono, Sanofi, Sumitomo, and Teijin. KMiyo reports a grant from Descente. KMiya reports to have been a member of a cooperative program between the University of Tokyo and NTT DOCOMO. TY reports grants and endowments from Asahi Mutual Life Insurance, Astellas, Bayer, Boehringer Ingelheim, EA, Eli Lilly, Kowa, Med Mirai, Mitsubishi Tanabe, MSD, Nipro, Nitto Boseki, Novo Nordisk, Ono, Shionogi, Sumitomo, Takeda, and Teijin; personal fees from Abbott, Arkray, Astellas, AstraZeneca, Bayer, Boehringer Ingelheim, Daiichi Sankyo, EA, Eli Lilly, Kowa, Kyowa Kirin, LifeScan, Medtronic, Mitsubishi Tanabe, MSD, Nipro, Nitto Boseki, Novartis, Novo Nordisk, Ono, Sanofi, Sanwa Kagaku Kenkyusho, Sumitomo, Taisho, Takeda, Teijin, Terumo, and Viatris. TK reports grants and endowments from Asahi Mutual Life Insurance (endowed course), Boehringer Ingelheim, Eli Lilly, Mitsubishi Tanabe, Sumitomo, and Taisho; personal fees from Abbott, Astellas, Boehringer Ingelheim, Eli Lilly, Fujifilm Toyama, Kowa, Mitsubishi Tanabe, MSD, Novo Nordisk, Ono, Sanofi, Sumitomo, Taisho, Teijin, and Terumo; a member of a data safety monitoring or advisory board of Eli Lilly and Novo Nordisk. The other authors have nothing to disclose."

Please confirm that this does not alter your adherence to all PLOS ONE policies on sharing data and materials, by including the following statement: "This does not alter our adherence to  PLOS ONE policies on sharing data and materials.” (as detailed online in our guide for authors http://journals.plos.org/plosone/s/competing-interests).  If there are restrictions on sharing of data and/or materials, please state these. Please note that we cannot proceed with consideration of your article until this information has been declared.

Please include your updated Competing Interests statement in your cover letter; we will change the online submission form on your behalf.

5. In this instance it seems there may be acceptable restrictions in place that prevent the public sharing of your minimal data. However, in line with our goal of ensuring long-term data availability to all interested researchers, PLOS’ Data Policy states that authors cannot be the sole named individuals responsible for ensuring data access (http://journals.plos.org/plosone/s/data-availability#loc-acceptable-data-sharing-methods).

Data requests to a non-author institutional point of contact, such as a data access or ethics committee, helps guarantee long term stability and availability of data. Providing interested researchers with a durable point of contact ensures data will be accessible even if an author changes email addresses, institutions, or becomes unavailable to answer requests.

Before we proceed with your manuscript, please also provide non-author contact information (phone/email/hyperlink) for a data access committee, ethics committee, or other institutional body to which data requests may be sent. If no institutional body is available to respond to requests for your minimal data, please consider if there any institutional representatives who did not collaborate in the study, and are not listed as authors on the manuscript, who would be able to hold the data and respond to external requests for data access? If so, please provide their contact information (i.e., email address). Please also provide details on how you will ensure persistent or long-term data storage and availability.

6. Please include captions for your Supporting Information files at the end of your manuscript, and update any in-text citations to match accordingly. Please see our Supporting Information guidelines for more information: http://journals.plos.org/plosone/s/supporting-information.

7. If the reviewer comments include a recommendation to cite specific previously published works, please review and evaluate these publications to determine whether they are relevant and should be cited. There is no requirement to cite these works unless the editor has indicated otherwise.

[Note: HTML markup is below. Please do not edit.]

Reviewers' comments:

Reviewer's Responses to Questions

**Comments to the Author**

1. Is the manuscript technically sound, and do the data support the conclusions?

Reviewer #1: Yes

Reviewer #2: Yes

Reviewer #3: Yes

Reviewer #4: Yes

Reviewer #5: Partly

**********

2. Has the statistical analysis been performed appropriately and rigorously?

Reviewer #1: Yes

Reviewer #2: Yes

Reviewer #3: Yes

Reviewer #4: Yes

Reviewer #5: Yes

**********

3. Have the authors made all data underlying the findings in their manuscript fully available?

The PLOS Data policy

Reviewer #1: Yes

Reviewer #2: Yes

Reviewer #3: Yes

Reviewer #4: Yes

Reviewer #5: No

**********

4. Is the manuscript presented in an intelligible fashion and written in standard English?

Reviewer #1: Yes

Reviewer #2: Yes

Reviewer #3: Yes

Reviewer #4: Yes

Reviewer #5: No

**********

Reviewer #1: Dear authors,

I am pleased to have the opportunity to review your manuscript, 'PONE-D-26-09852'. Your study addresses a critical gap in diabetes management by re-evaluating HbA1c targets in a contemporary clinical environment in which severe hypoglycaemia is uncommon. The use of updated mean values and the landmark analysis approach are both statistically sound, providing a dynamic view of risk factor management. However, to strengthen the clinical applicability and robustness of your findings further, I have the following comments and requests for additional analysis:

1. The study population predominantly consists of participants for primary prevention, with only 11.4% having a history of cardiovascular disease. Although baseline cardiovascular disease (CVD) history was used as a covariate in your Cox regression models, the risk structures for primary and secondary prevention are fundamentally different. Given that primary prevention represents the vast majority of your cohort, please perform a sensitivity analysis extracting only the primary prevention group and confirm that the tendency for all-cause mortality and myocardial infarction remains consistent within this subgroup as it does in the total population.

2. Your analysis begins at the landmark time point of 1 year after baseline. This approach may introduce a 'healthy survivor bias', as participants who experienced events or discontinued treatment early on are excluded. Please discuss how the exclusion of these early-phase cases might have influenced the observed hazard ratios.

3. The J-DOIT3 study period coincided with an increased use of newer agents, such as SGLT2 inhibitors and GLP-1 receptor agonists. It is unclear whether improved outcomes are solely attributable to the numerical reduction in HbA1c, or whether the pleiotropic effects of specific drug classes also played a role. Please address the potential confounding effect of medication types on your results.

4. The conclusion states that there is no clear lower threshold for HbA1c based on the lowest category of <6.5%. As this lowest category was not further subdivided, the assertion that 'no lower threshold exists' should be carefully qualified. Please discuss the limitations of the data in defining an absolute lower limit.

5. HDL-C had a notable impact on different outcomes in men and women. Please provide a more detailed biological or clinical explanation as to why the protective role of HDL-C appears to be sex-specific in your cohort.

Reviewer #2: The team intended to identify the thresholds of HbA1c and other clinical determinants associated with a minimized risk of macrovascular complications of diabetes in the setting of the J-DOIT3 trial.

1. How the participants were assigned need clarification. For example, how to implement based on age (exact match?) and so on?

2. The measures after one year landmark time point were collected which implies the exclusion of early outcome estimation. Is there any early treatment effects or risk?

3. Cox regression was implemented. Is the proportional hazard assumption validated?

4. While the sample size is large, some of events are small, e.g., n= 16 for MI. Please comment on the robustness of the reported results and the power for the analysis.

5. 75 participants were excluded from the analysis. Please comment on whether these may lead to any biased results.

6. Threshold analysis with stratified analysis in Table 4 is concerning, especially with small number of events. Also, comment on NA results! Also, please comment on how these thresholds were determined

7. Interpretation on the not significant results should be more cautious as the power may be limited for some of these analysis

Reviewer #3: The manuscript is well written, and the findings are clearly presented with adequate supporting data. The paper is well organized and written in standard English. Overall, the study is methodologically sound, and I recommend it for publication.

Reviewer #4: This study is a sub-analysis to verify the optimal management target values for each individual risk factor in the J-DOIT3 study. The results obtained are very interesting, but the authors need to reconsider the following points.

1. The J-DOIT3 study also evaluated renal outcomes, but why were they not included in this analysis?

2. To improve the visual clarity of research results and enhance reader comprehension, forest plots are preferable to tables.

3. What are the optimal values for BMI and triglycerides to prevent cardiovascular events?

4. The abstract must be no more than 300 words, so it needs to be kept short to comply with the requirements.

Reviewer #5: Dear Authors,

This manuscript addresses an important subject by considering the use of new drugs for diabetes treatment associated with fewer episodes of severe hypoglycemia and the incidence of macrovascular complications. Despite the limitations of an open-label study, it provides data that would help establish target levels of HbA1C, SBP and LDL-c to prevent macrovascular complications. However, it is necessary to make some revisions and clarifications described below.

#1 It is necessary to make grammatical and punctuation corrections in the whole manuscript.

#2 The methods deserve clarification.

- The study describes 2 groups of participants with different treatment targets, and Table 1 shows baseline data for a landmark point, which appears to be the data reported when all participants were already included in the study, but it is not very clear.

- It also says that participants' data from the year after that landmark point would be in the supplementary material (Table 2). Supplementary Table 2 shows the combined data for the 2 groups, and the mean levels of HbA1C, LDL, and SBP are outside the pre-established targets for both groups. It is important to report the data for each group separately, as well as the distributions of the number of patients who achieved, stratified by the analysis stratification shown in the results.

#3 It is worth including in the discussion comments on the real differences or similarities between the 2 groups and the combined analysis, which may affect the conclusions.

**********

what does this mean?). If published, this will include your full peer review and any attached files.

If you choose “no”, your identity will remain anonymous but your review may still be made public.

**Do you want your identity to be public for this peer review?**  For information about this choice, including consent withdrawal, please see our Privacy Policy

Reviewer #1: **Yes:**  KYUYONG CHO

Reviewer #2: No

Reviewer #3: **Yes:**  Dr. Momtaz Ahmed

Head of National Diabetes Centre, Suva, Fiji.

Senior Fellow International Diabetes Federation

Reviewer #4: **Yes:**  Ken-ichi Aihara

Reviewer #5: No

**********

[NOTE: If reviewer comments were submitted as an attachment file, they will be attached to this email and accessible via the submission site. Please log into your account, locate the manuscript record, and check for the action link "View Attachments". If this link does not appear, there are no attachment files.]

To ensure your figures meet our technical requirements, please review our figure guidelines: https://journals.plos.org/plosone/s/figures

You may also use PLOS’s free figure tool, NAAS, to help you prepare publication quality figures: https://journals.plos.org/plosone/s/figures#loc-tools-for-figure-preparation.

NAAS will assess whether your figures meet our technical requirements by comparing each figure against our figure specifications.

# Author comment: Author response to Decision Letter 1

**DOI:** 10.1371/journal.pone.0360295.r002

10 Jun 2026

The following are the authors’ responses to the comments from the editor and reviewers.

From and To the Editor

1. The small number of outcome events, particularly myocardial infarction (n = 16), represents a critical limitation. The stratified threshold analyses presented in Table 4, which underpin your central recommendation of an HbA1c target of <6.5%, are considered statistically weak. You are required to reframe non-significant findings with appropriate caution, explicitly acknowledging the inability to distinguish true null effects from type II errors. Furthermore, instances yielding "NA" results due to insufficient events must be accompanied by discussion rather than left without comment.

Response:

We appreciate your thoughtful feedback. Based on your comments, we have made the following revisions to tone down the wording. We deleted “Rather, it appeared to increase from the lowest to the highest HbA1c category, especially for all-cause mortality and myocardial infarction, suggesting the absence of any J curve-like association. (lines 407-409; in a marked-up copy hereafter)” Instead, we added the following. “However, we should be very cautious, especially regarding myocardial infarction, because statistically significant differences in the second and third categories might have been overlooked due to the small number of events. (lines 410-412).”

In addition, we have also revised the relevant sections in the "Conclusions" of the ABSTRACT, and in the second and final paragraphs of the "DISCUSSION" section.

We also added a limitation stating that among women, we were unable to fully perform an analysis of the association between HDL-cholesterol and myocardial infarction, as well as a part of stroke, due to the small number of events (lines 469-471), and provided the specific numbers of events as a footnote of this table.

2. The 1-year landmark design introduces a potential healthy survivor bias, as participants experiencing early events or discontinuing treatment prior to the landmark were excluded. The exclusion of 75 participants due to missing variables also requires explicit justification. Please provide relevant sensitivity analyses and a thorough discussion of how these exclusions may have affected the hazard ratio estimates.

Response:

We conducted an analysis using updated mean values in order to assess the direct effects of treatment during the intervention period. Furthermore, to minimize the influence of pre-registration treatment as much as possible, we attempted landmark analysis with a landmark time of 1-year that we assumed to be adequate and employed this term. Given the nature of this landmark analysis, which requires values from the first term, we performed a sensitivity analysis using a 6-month (180-day) landmark time-point (with 2507 participants) instead of 1 year. However, although there was one death in the latter half of the first year, the data for the variables of this participant were missing, and it was not included in the analysis; there were no cases of myocardial infarction during this period. Therefore, the results remained virtually unchanged (the changes in those values were by far smaller than the smallest digit in the table) for both of these two outcomes. With regard to stroke, seven cases with no missing data were identified during this period; the results are presented in Supplemental Table 4 (re-numbered). Alteration of the landmark time-point did not show a substantial difference in the stroke, either. We have added this information to the RESULTS section (lines 352-360).

In addition, regarding the 75 participants excluded due to missing data for variables when using the landmark time-point of 1 year, we have specified the details and included this information in the RESULTS section (lines 290-293).

3. The statement that there is "no lower threshold for HbA1c," and the resulting recommendation of a <6.5% target, is not adequately supported by the data. Given that the <6.5% category was not further subdivided and that confidence intervals are extremely wide, the absolute lower limit of safe glycemic control cannot be established from this dataset alone. The authors are advised to qualify this conclusion.

Response:

We have deleted the part of “no lower threshold for HbA1c”. Instead, we added a phrase “although given that the category of <6.5% has not been subdivided and the confidence interval is wide, our analysis does not identify the absolute lower limit for safe blood glucose control.” (lines 461-463)

4. The manuscript does not sufficiently address the possibility that observed outcome improvements reflect the pleiotropic cardiovascular effects of specific drug classes, such as SGLT2 inhibitors and GLP-1 receptor agonists, rather than HbA1c reduction per se. Additionally, the omission of renal outcomes, BMI, and triglyceride levels from the analyses requires justification, as these represent clinically relevant covariates whose absence limits the completeness of the risk factor evaluation.

Response:

This study was completed in March 2016. At that time-point, below 5% of patients had been prescribed GLP-1 receptor agonists at the final visit in both the intensive and conventional therapy groups, which was also the case with SGLT2 inhibitors, as shown in the Supplementary table 2 of the ref. #8 of the manuscript; therefore, it is unlikely that this had a significant impact on the results. We added this sentence to the DISCUSSION section. (lines 482-487)

Renal outcomes have been separated out as microvascular events and have been discussed in detail in a separate paper already published (Kidney Int. 2021 Jan;99(1):256-266).

The purpose of the current analysis was to identify factors mediating the effects of intervention of the J-DOIT3; because HDL cholesterol levels showed a significant difference between the intensive and conventional therapy groups, as shown in Figure 2 of the main analysis paper (ref. #8 of the manuscript), this variable was included in the analysis. Since there was only a small difference in BMI trends between the two groups, as shown in the same figure, it was not included as an analytical variable. Regarding triglycerides, since we included HDL-cholesterol as described above, triglycerides were omitted, considering the correlation between the two. This was also the case with the procedure that only systolic blood pressure was employed, rather than both systolic and diastolic blood pressure. These sentences are now included (below Table 3).

From and To the Reviewer #1:

I am pleased to have the opportunity to review your manuscript, 'PONE-D-26-09852'. Your study addresses a critical gap in diabetes management by re-evaluating HbA1c targets in a contemporary clinical environment in which severe hypoglycaemia is uncommon. The use of updated mean values and the landmark analysis approach are both statistically sound, providing a dynamic view of risk factor management.

However, to strengthen the clinical applicability and robustness of your findings further, I have the following comments and requests for additional analysis:

Response:

We greatly appreciate your encouraging comment above.

Regarding the points you raised, our response is as follows.

1. The study population predominantly consists of participants for primary prevention, with only 11.4% having a history of cardiovascular disease. Although baseline cardiovascular disease (CVD) history was used as a covariate in your Cox regression models, the risk structures for primary and secondary prevention are fundamentally different. Given that primary prevention represents the vast majority of your cohort, please perform a sensitivity analysis extracting only the primary prevention group and confirm that the tendency for all-cause mortality and myocardial infarction remains consistent within this subgroup as it does in the total population.

Response:

Thank you very much for your thoughtful suggestion. We have performed your proposal as a sensitivity analysis. The result is now included in Supplemental Table 7, and the tendency for all-cause mortality and myocardial infarction remains basically consistent within this sub-cohort to that in the total population. We have added this information in the last part of the RESULTS section (lines 372-376).

2. Your analysis begins at the landmark time point of 1 year after baseline. This approach may introduce a 'healthy survivor bias', as participants who experienced events or discontinued treatment early on are excluded. Please discuss how the exclusion of these early-phase cases might have influenced the observed hazard ratios.

Response:

Thank you very much for your important comment. Regarding this point, please refer to our responses to the Editor’s Comment 2.

3. The J-DOIT3 study period coincided with an increased use of newer agents, such as SGLT2 inhibitors and GLP-1 receptor agonists. It is unclear whether improved outcomes are solely attributable to the numerical reduction in HbA1c, or whether the pleiotropic effects of specific drug classes also played a role. Please address the potential confounding effect of medication types on your results.

Response:

Thank you very much for your valuable feedback. On this point, please refer to our response to the Editor’s comment 4.

4. The conclusion states that there is no clear lower threshold for HbA1c based on the lowest category of <6.5%. As this lowest category was not further subdivided, the assertion that 'no lower threshold exists' should be carefully qualified. Please discuss the limitations of the data in defining an absolute lower limit.

Response:

We appreciate your pointing out this. Regarding this issue, please refer to our response to the Editor’s comment 3.

5. HDL-C had a notable impact on different outcomes in men and women.

Please provide a more detailed biological or clinical explanation as to why the protective role of HDL-C appears to be sex-specific in your cohort.

Thank you for your insightful comment. Inspired by your suggestion, we have incorporated the following points into the discussion (lines 447-451) and added two papers to reference (re-numbered #29 & #30).

“Although it is difficult to draw a precise picture regarding gender differences in the correlation between stroke and HDL cholesterol, and between all-cause mortality and HDL cholesterol, our analysis may be in line with the findings that, in the general Japanese population, the correlation with HDL cholesterol tends to be stronger for all-cause mortality among women, and for stroke among men.”

From and To the Reviewer #2:

The team intended to identify the thresholds of HbA1c and other clinical determinants associated with a minimized risk of macrovascular complications of diabetes in the setting of the J-DOIT3 trial.

Response:

We greatly appreciate your insightful expression above.

1. How the participants were assigned need clarification. For example, how to implement based on age (exact match?) and so on?

Response:

We are very grateful if you could kindly refer to the design paper (ref. #9 of the manuscript) as this information is included therein.

2. The measures after one year landmark time point were collected which implies the exclusion of early outcome estimation. Is there any early treatment effects or risk?

Response:

Thank you very much for your valuable comment. Regarding this point, please refer to our responses to the Editor’s Comment 2.

3. Cox regression was implemented. Is the proportional hazard assumption validated?

Response:

The proportional hazards assumption for the treatment group was assessed by visual inspection of log-minus-log survival plots. We acknowledge that, in randomized controlled trials, treatment effects may vary over time and strict proportionality may not always hold throughout the entire follow-up period.

To address this issue, we additionally evaluated models incorporating time-dependent covariates and treatment-by-covariate interaction terms in this report. The inclusion of time-dependent covariates and interaction terms was intended to account for potential non-proportionality and to evaluate the robustness of the findings under less restrictive modeling assumptions. These analyses yielded results that were materially unchanged from the primary analysis of J-DOIT3, supporting the robustness of the reported treatment effect.

4. While the sample size is large, some of events are small, e.g., n= 16 for MI. Please comment on the robustness of the reported results and the power for the analysis.

Response:

We appreciate your important comments. We have toned down our claims regarding the result of myocardial infarction, given the small number of events. Please refer to our responses to the Editor’s Comment 1 regarding this point. We have also conducted sensitivity analyses to assess the robustness of the results, one as mentioned above (Response #2), and we also extracted and analyzed only participants with no history of cardiovascular events—that is, the primary prevention cohort (Supplementary Table 7) as another sensitivity analysis.

5. 75 participants were excluded from the analysis. Please comment on whether these may lead to any biased results.

Response:

As you pointed out, we excluded 75 participants due to insufficient data for adjustment. Regarding these 75 participants, we newly described the specific breakdown is in lines 290-293. Because of this, we are not able to rule out the possibility that this might have introduced some bias. This point is now mentioned as the fourth limitation (lines 473- 475).

6. Threshold analysis with stratified analysis in Table 4 is concerning, especially with small number of events. Also, comment on NA results!

Also, please comment on how these thresholds were determined

Response:

We appreciate your comments very much. We have toned down our claims regarding the result of myocardial infarction, given the small number of events. Regarding this point, please refer to our responses to the Editor’s Comment 1.

We also added a limitation stating that among women, we were unable to fully perform an analysis of the association between HDL-cholesterol and myocardial infarction, as well as a part of stroke, due to the small number of events (lines 469-471), and provided the specific numbers of events as a footnote of Table 4.

7. Interpretation on the not significant results should be more cautious as the power may be limited for some of these analysis

Response:

Thank you for your important comment. With regard to this point, we have made the following revisions to tone down the wording. We deleted a sentence (lines 407-409) and, instead, we have added the following. “However, we should be very cautious, especially regarding myocardial infarction, because statistically significant differences in the second and third categories might have been overlooked due to the small number of events. (lines 410-412).”

From and To the Reviewer #3:

The manuscript is well written, and the findings are clearly presented with adequate supporting data. The paper is well organized and written in standard English. Overall, the study is methodologically sound, and I recommend it for publication.

Response:

Thank you very much for your kind comments on the significance, content, and methodology of my paper. We are deeply grateful for your recommendation for publication.

From and To the Reviewer #4:

This study is a sub-analysis to verify the optimal management target values for each individual risk factor in the J-DOIT3 study. The results obtained are very interesting, but the authors need to reconsider the following points.

Response:

Thank you for your feedback and advice. Based on your comments, we have revised the manuscript as follows.

1. The J-DOIT3 study also evaluated renal outcomes, but why were they not included in this analysis?

Response:

Renal outcomes have been separated out as microvascular events and have been discussed in detail in a separate paper already published (Kidney Int. 2021 Jan;99(1):256-266).

2. To improve the visual clarity of research results and enhance reader comprehension, forest plots are preferable to tables.

Response:

As is common in epidemiological studies, we used tables rather than forest plots. We believe that tables provide information essential to scientific evidence, such as “detailed data disclosure,” “comprehensive comparative analysis,” and “presentation of results adjusted for confounding factors.”

3. What are the optimal values for BMI and triglycerides to prevent cardiovascular events?

Response:

The purpose of the current analysis was to identify factors mediating the effects of intervention of the J-DOIT3; because HDL cholesterol levels showed a significant difference between the intensive and conventional therapy groups, as shown in Figure 2 of the main analysis paper (ref. #8 of the manuscript), this variable was included in the analysis. Since there was only a small difference in BMI trends between the two groups, as shown in the same figure, it was not included as an analytical variable. Regarding triglycerides, since we included HDL-cholesterol as described above, triglycerides were omitted, considering the correlation between the two. This was also the case with the procedure that only systolic blood pressure was employed, rather than both systolic and diastolic blood pressure. These sentences are now included (below Table 3).

4. The abstract must be no more than 300 words, so it needs to be kept short to comply with the requirements.

Response:

According to your advice, we have deleted more than one phrase of a sentence of the abstract, and now the word count is 299 except section titles and fundings.

From and To the Reviewer #5:

Dear Authors,

This manuscript addresses an important subject by considering the use of new drugs for diabetes treatment associated with fewer episodes of severe hypoglycemia and the incidence of macrovascular complications.

Despite the limitations of an open-label study, it provides data that would help establish target levels of HbA1C, SBP and LDL-c to prevent macrovascular complications. However, it is necessary to make some revisions and clarifications described below.

Response:

We greatly appreciate your comments, right to the point, and we have amended the manuscript according to your queries.

#1 It is necessary to make grammatical and punctuation corrections in the whole manuscript.

Response:

Thank you very much. Based on your advice, we have reviewed and amended the grammatical errors throughout the entire text.

#2 The methods deserve clarification.

- The study describes 2 groups of participants with different treatment targets, and Table 1 shows baseline data for a landmark point, which appears to be the data reported when all participants were already included in the study, but it is not very clear.

Response:

Table 1 presents data for all participants one year after enrollment. To clarify this point, a new sentence has been added at the beginning of the footnote.

- It also says that participants' data from the year after that landmark point would be in the supplementary material (Table 2). Supplementary Table 2 shows the combined data for the 2 groups, and the mean levels of HbA1C, LDL, and SBP are outside the pre-established targets for both groups. It is important to report the data for each group separately, as well as the distributions of the number of patients who achieved, stratified by the analysis stratification shown in the results.

Response:

The trends in the data for each group are presented in the main analysis paper (ref. #8); in this table, we intended to present the data used for the combined landmark analysis of both groups.

#3 It is worth including in the discussion comments on the real differences or similarities between the 2 groups and the combined analysis, which may affect the conclusions.

Response:

Although the policy to combine the two groups for analysis is noted at the beginning of the Results section, we also reiterated this point in the first paragraph of the DISCUSSION section (lines 400-404).

Other than the above-mentioned, we have adjusted the tables’ formatting, but the content has not been changed. Also, the name of the institution where one of the authors is affiliated has been changed, so this has been updated accordingly. Also, affiliation change for a new physical year in Japan We have also updated the information to reflect changes in the affiliated institution that commenced with the new fiscal year in Japan beginning in April.

Attachment Submitted filename: Response_Ver18_cleaned.docx

# Peer review: Decision Letter 1

**DOI:** 10.1371/journal.pone.0360295.r003

**Authors:** Hidetaka Hamasaki

24 Jun 2026

Dear Dr. Sasako,

Thank you for submitting your manuscript to PLOS ONE. After careful consideration, we feel that it has merit but does not fully meet PLOS ONE’s publication criteria as it currently stands. Therefore, we invite you to submit a revised version of the manuscript that addresses the points raised during the review process.

Please submit your revised manuscript by Aug 08 2026 11:59PM. If you will need more time than this to complete your revisions, please reply to this message or contact the journal office at plosone@plos.org. When you're ready to submit your revision, log on to https://www.editorialmanager.com/pone/ and select the 'Submissions Needing Revision' folder to locate your manuscript file.

- A letter that responds to each point raised by the academic editor and reviewer(s). You should upload this letter as a separate file labeled 'Response to Reviewers'.
- A marked-up copy of your manuscript that highlights changes made to the original version. You should upload this as a separate file labeled 'Revised Manuscript with Track Changes'.
- An unmarked version of your revised paper without tracked changes. You should upload this as a separate file labeled 'Manuscript'.

If you would like to make changes to your financial disclosure, please include your updated statement in your cover letter. Guidelines for resubmitting your figure files are available below the reviewer comments at the end of this letter.

If applicable, we recommend that you deposit your laboratory protocols in protocols.io to enhance the reproducibility of your results. Protocols.io assigns your protocol its own identifier (DOI) so that it can be cited independently in the future. For instructions see: https://journals.plos.org/plosone/s/submission-guidelines#loc-laboratory-protocols. Additionally, PLOS ONE offers an option for publishing peer-reviewed Lab Protocol articles, which describe protocols hosted on protocols.io. Read more information on sharing protocols at https://plos.org/protocols?utm_medium=editorial-email&utm_source=authorletters&utm_campaign=protocols.

As the corresponding author, your ORCID iD is verified in the submission system and will appear in the published article. PLOS supports the use of ORCID, and we encourage all coauthors to register for an ORCID iD and use it as well. Please encourage your coauthors to verify their ORCID iD within the submission system before final acceptance, as unverified ORCID iDs will not appear in the published article. *Only*  the individual author can complete the verification step; PLOS staff *cannot*  verify ORCID iDs on behalf of authors.

We look forward to receiving your revised manuscript.

Kind regards,

Hidetaka Hamasaki

Academic Editor

PLOS One

Journal Requirements:

1. If the reviewer comments include a recommendation to cite specific previously published works, please review and evaluate these publications to determine whether they are relevant and should be cited. There is no requirement to cite these works unless the editor has indicated otherwise.

2. Please review your reference list to ensure that it is complete and correct. If you have cited papers that have been retracted, please include the rationale for doing so in the manuscript text, or remove these references and replace them with relevant current references. Any changes to the reference list should be mentioned in the rebuttal letter that accompanies your revised manuscript. If you need to cite a retracted article, indicate the article’s retracted status in the References list and also include a citation and full reference for the retraction notice.

[Note: HTML markup is below. Please do not edit.]

Reviewers' comments:

Reviewer's Responses to Questions

**Comments to the Author**

Reviewer #2: All comments have been addressed

Reviewer #4: (No Response)

Reviewer #5: All comments have been addressed

**********

2. Is the manuscript technically sound, and do the data support the conclusions?

Reviewer #2: (No Response)

Reviewer #4: Yes

Reviewer #5: Partly

**********

3. Has the statistical analysis been performed appropriately and rigorously?

Reviewer #2: (No Response)

Reviewer #4: Yes

Reviewer #5: Yes

**********

4. Have the authors made all data underlying the findings in their manuscript fully available?

The PLOS Data policy

Reviewer #2: (No Response)

Reviewer #4: Yes

Reviewer #5: Yes

**********

5. Is the manuscript presented in an intelligible fashion and written in standard English?

Reviewer #2: (No Response)

Reviewer #4: Yes

Reviewer #5: Yes

**********

Reviewer #2: (No Response)

Reviewer #4: Although there are some differences of opinion between the authors and the reviewer, I would like to comment on the following points.

1. The authors have stated that they have already published findings on the renal outcomes of the J-DOIT3 study (Kidney Int. 2021. Jan;99(1):256-266), but this should be mentioned in the text and included in the references. It should be noted that not all readers are familiar with the J-DOIT3 study.

2. Forest plots can adequately represent the adjustment for confounding factors and provide sufficient scientific evidence. My opinion remains that they are a useful method for busy readers to quickly and visually grasp the outline of the research, but I respect the authors' decision to present the information in a table.

Reviewer #5: I am glad to rereview this manuscript. The authors addressed most of the reviewers' comments and added some important observations. However, considering the data from your previous study, only part of the participants in the study achieved the pre-established goals of treatment in both groups, especially in the intensive treatment group where only 18% achived HbA1C below 6.2%, with a mean level of 6,8%( reference data) ; it is still considered important to make this clear in the discussion, as the low incidence of severe hypoglycemia may be affected by such data, and strict control could possibly affect the number of events and the significance of the results related to the safety of lower levels of HbA1C.

**********

what does this mean?). If published, this will include your full peer review and any attached files.

If you choose “no”, your identity will remain anonymous but your review may still be made public.

**Do you want your identity to be public for this peer review?**  For information about this choice, including consent withdrawal, please see our Privacy Policy

Reviewer #2: No

Reviewer #4: **Yes:**  Ken-ichi Aihara

Reviewer #5: No

**********

[NOTE: If reviewer comments were submitted as an attachment file, they will be attached to this email and accessible via the submission site. Please log into your account, locate the manuscript record, and check for the action link "View Attachments". If this link does not appear, there are no attachment files.]

To ensure your figures meet our technical requirements, please review our figure guidelines: https://journals.plos.org/plosone/s/figures

You may also use PLOS’s free figure tool, NAAS, to help you prepare publication quality figures: https://journals.plos.org/plosone/s/figures#loc-tools-for-figure-preparation.

NAAS will assess whether your figures meet our technical requirements by comparing each figure against our figure specifications.

# Author comment: Author response to Decision Letter 2

**DOI:** 10.1371/journal.pone.0360295.r004

28 Aug 2026

The following are the authors’ responses to the comments from the editor and reviewers.

From and To the Reviewer #4:

Although there are some differences of opinion between the authors and the reviewer, I would like to comment on the following points.

Response:

Thank you for your important suggestions. Based on your comments, we have revised the manuscript as follows.

1. The authors have stated that they have already published findings on the renal outcomes of the J-DOIT3 study (Kidney Int. 2021. Jan;99(1):256-266), but this should be mentioned in the text and included in the references. It should be noted that not all readers are familiar with the J-DOIT3 study.

Response:

Thank you very much for your constructive and receptive comment. We have added the following text as the final paragraph of the “Introduction” section (lines 131-133), citing the relevant reference (#16) that you mentioned.

“It should be noted here that detailed results and discussions regarding renal outcomes—which are no less important than cardiovascular outcomes in diabetic vascular complications—have already been reported in our previous publication.16”

2. Forest plots can adequately represent the adjustment for confounding factors and provide sufficient scientific evidence. My opinion remains that they are a useful method for busy readers to quickly and visually grasp the outline of the research, but I respect the authors' decision to present the information in a table.

Response:

Thank you for your valuable insights and for accepting our approach on this matter. We think each of these approaches has its own advantages, and we imagine you would probably share this view.

From and To the Reviewer #5:

I am glad to rereview this manuscript. The authors addressed most of the reviewers' comments and added some important observations.

Response:

Thank you very much for your kind words. We, the authors, are truly encouraged by your positive feedback.

However, considering the data from your previous study, only part of the participants in the study achieved the pre-established goals of treatment in both groups, especially in the intensive treatment group where only 18% achived HbA1C below 6.2%, with a mean level of 6,8%( reference data) ; it is still considered important to make this clear in the discussion, as the low incidence of severe hypoglycemia may be affected by such data, and strict control could possibly affect the number of events and the significance of the results related to the safety of lower levels of HbA1C.

Response:

We appreciate your more detailed and specific important comment. In response to your comment, we have amended the manuscript by adding a cohesive pair of sentences as the fifth limitation as follows (lines 463-468). Moreover, in connection with this, we have toned down the nuance of the final part of the “discussion” section (lines 481-482).

“Fifth, in the J-DOIT3 study, only a part of participants in both groups achieved the pre-specified HbA1c treatment targets; in particular, in the intensive therapy group, the mean HbA1c during the intervention period was 6.8%, and only 18% achieved an HbA1c level below the target of 6.2%. This may have masked the occurrence of events at further lower HbA1c levels, potentially resulting in insufficient evaluation of HbA1c at those levels. It is crucial to note that this analysis does not guarantee safety at very low HbA1c levels.”

Attachment Submitted filename: Response (re-revision)04.docx

# Peer review: Decision Letter 2

**DOI:** 10.1371/journal.pone.0360295.r005

**Authors:** Hidetaka Hamasaki

26 Sep 2026

Search for the Glycated Hemoglobin Target Minimizing Mortality among People with Type 2 Diabetes with Rare Incidence of Treatment-related Severe Hypoglycemia – Epidemiologic Analysis of J-DOIT3 Randomized Controlled Trial

PONE-D-26-09852R2

Dear Dr. Sasako,

We’re pleased to inform you that your manuscript has been judged scientifically suitable for publication and will be formally accepted for publication once it meets all outstanding technical requirements.

Within one week, you’ll receive an e-mail detailing the required amendments. When these have been addressed, you’ll receive a formal acceptance letter and your manuscript will be scheduled for publication.

An invoice will be generated when your article is formally accepted. Please note, if your institution has a publishing partnership with PLOS and your article meets the relevant criteria, all or part of your publication costs will be covered. Please make sure your user information is up-to-date by logging into Editorial Manager at Editorial Manager® and clicking the ‘Update My Information' link at the top of the page. For questions related to billing, please contact billing support.

If your institution or institutions have a press office, please notify them about your upcoming paper to help maximize its impact. If they’ll be preparing press materials, please inform our press team as soon as possible -- no later than 48 hours after receiving the formal acceptance. Your manuscript will remain under strict press embargo until 2 pm Eastern Time on the date of publication. For more information, please contact onepress@plos.org.

Kind regards,

Hidetaka Hamasaki

Academic Editor

PLOS One

Additional Editor Comments (optional):

Reviewers' comments:

Reviewer's Responses to Questions

**Comments to the Author**

Reviewer #2: (No Response)

Reviewer #4: All comments have been addressed

Reviewer #5: All comments have been addressed

**********

2. Is the manuscript technically sound, and do the data support the conclusions?

Reviewer #2: (No Response)

Reviewer #4: Yes

Reviewer #5: Yes

**********

3. Has the statistical analysis been performed appropriately and rigorously?

Reviewer #2: (No Response)

Reviewer #4: Yes

Reviewer #5: Yes

**********

4. Have the authors made all data underlying the findings in their manuscript fully available?

The PLOS Data policy

Reviewer #2: (No Response)

Reviewer #4: No

Reviewer #5: Yes

**********

5. Is the manuscript presented in an intelligible fashion and written in standard English?

Reviewer #2: (No Response)

Reviewer #4: Yes

Reviewer #5: Yes

**********

Reviewer #2: (No Response)

Reviewer #4: In the revised manuscript, I have no further comment regarding the authors' responses for my concerns.

Reviewer #5: (No Response)

**********

what does this mean?). If published, this will include your full peer review and any attached files.

If you choose “no”, your identity will remain anonymous but your review may still be made public.

**Do you want your identity to be public for this peer review?**  For information about this choice, including consent withdrawal, please see our Privacy Policy

Reviewer #2: No

Reviewer #4: **Yes:**  Ken-ichi Aihara

Reviewer #5: No

**********

# Editor report: Acceptance letter

**DOI:** 10.1371/journal.pone.0360295.r006

**Authors:** Hidetaka Hamasaki

PONE-D-26-09852R2

PLOS One

Dear Dr. Sasako,

I'm pleased to inform you that your manuscript has been deemed suitable for publication in PLOS One. Congratulations! Your manuscript is now being handed over to our production team.

At this stage, our production department will prepare your paper for publication. This includes ensuring the following:

* All references, tables, and figures are properly cited

* All relevant supporting information is included in the manuscript submission,

* There are no issues that prevent the paper from being properly typeset

You will receive further instructions from the production team, including instructions on how to review your proof when it is ready. Please keep in mind that we are working through a large volume of accepted articles, so please give us a few days to review your paper and let you know the next and final steps.

Lastly, if your institution or institutions have a press office, please let them know about your upcoming paper now to help maximize its impact. If they'll be preparing press materials, please inform our press team within the next 48 hours. Your manuscript will remain under strict press embargo until 2 pm Eastern Time on the date of publication. For more information, please contact onepress@plos.org.

You will receive an invoice from PLOS for your publication fee after your manuscript has reached the completed accept phase. If you receive an email requesting payment before acceptance or for any other service, this may be a phishing scheme. Learn how to identify phishing emails and protect your accounts at https://explore.plos.org/phishing.

If we can help with anything else, please email us at customercare@plos.org.

Thank you for submitting your work to PLOS One and supporting open access.

Kind regards,

PLOS One Editorial Office Staff

on behalf of

Dr. Hidetaka Hamasaki

Academic Editor

PLOS One
